# Translation error clusters induced by aminoglycoside antibiotics

**DOI:** 10.1038/s41467-021-21942-6

**Published:** 2021-03-23

**Authors:** Ingo Wohlgemuth, Raffaella Garofalo, Ekaterina Samatova, Aybeg Nafiz Günenç, Christof Lenz, Henning Urlaub, Marina V. Rodnina

**Affiliations:** 1 Department of Physical Biochemistry, Max Planck Institute for Biophysical Chemistry, Goettingen, Germany; 2 Bioanalytical Mass Spectrometry Group, Max Planck Institute for Biophysical Chemistry, Goettingen, Germany; 3 Institute of Clinical Chemistry, Bioanalytics, University Medical Center Goettingen, Goettingen, Germany

**Keywords:** Mass spectrometry, Mechanism of action, Antibiotics, Ribosome

## Abstract

Aminoglycoside antibiotics target the ribosome and induce mistranslation, yet which translation errors induce bacterial cell death is unclear. The analysis of cellular proteins by quantitative mass spectrometry shows that bactericidal aminoglycosides induce not only single translation errors, but also clusters of errors in full-length proteins in vivo with as many as four amino acid substitutions in a row. The downstream errors in a cluster are up to 10,000-fold more frequent than the first error and independent of the intracellular aminoglycoside concentration. The prevalence, length, and composition of error clusters depends not only on the misreading propensity of a given aminoglycoside, but also on its ability to inhibit ribosome translocation along the mRNA. Error clusters constitute a distinct class of misreading events in vivo that may provide the predominant source of proteotoxic stress at low aminoglycoside concentration, which is particularly important for the autocatalytic uptake of the drugs.

## Abstract (web summary)

Aminoglycoside antibiotics target the ribosome and induce misreading, yet which translation errors induce bacterial cell death is unclear. Here authors use quantitative mass spectrometry and show that bactericidal aminoglycosides induce clusters of errors in full-length proteins in vivo with as many as four amino acid substitutions in a row.

## Introduction

Aminoglycoside antibiotics (AGAs) are positively charged oligosaccharides that target bacterial ribosomes. AGAs induce misreading of the mRNA, resulting in the incorporation of incorrect amino acids into protein, and/or inhibit translocation, the movement of the ribosome along the mRNA^1–3^. Many AGAs, such as paromomycin (Par), tobramycin (Tob), neomycin (Neo), gentamicin (Gen), kanamycin (Kan), or amikacin (Amk), are clinically important as broad-spectrum antibiotics. On the other hand, their propensity to induce translation errors can be utilized to alleviate the symptoms of human genetic diseases (e.g., Duchenne muscular dystrophy) by increasing readthrough of premature stop codons^4^. The negative side effect of AGA treatment is its unfavorable effect on translation in mitochondria^5–7^. In particular, the high oto- and nephrotoxicity due to targeting of mitoribosomes often hampers systemic administration of AGAs^8^.

Although misreading is the key element of the AGA action, we know surprisingly little which types of errors are induced and how they affect the proteome and the fitness of bacterial cells. AGAs preferentially induce a subset of errors that result from a misreading of the third position of the mRNA codon, but errors in the first and second position can also occur^9–11^. The type of most prevalent errors depends on the drug, e.g., streptomycin (Str) is less efficient than other AGAs in inducing U-G mismatches in the second position or G-U mismatches at any position of the codon–anticodon complexes^10^. Severe mistranslation leads to growth arrest or cell death in bacteria^12–15^. However, the AGA-induced error load alone cannot explain their bactericidal effects. In fact, ribosomal ambiguity (*ram*) mutants, with alterations in ribosomal protein S4, also cause increased misreading, but grow at almost wild-type (*wt*) rates^16^. This led to the notion that there is something particular to the bactericidal effect of AGAs^17,18^, but what causes their detrimental effect on bacterial fitness and cell viability remains unclear.

AGAs are polycationic molecules that bind to the negatively charged outer membrane of bacteria and enter the periplasm via porin channels. Once in the cytosol, AGAs bind to the ribosome and induce misreading by stabilizing an error-prone conformation of the decoding center of the ribosome^19–21^. Accumulation of translation errors in membrane proteins leads to the disintegration of membrane structures, renders the membrane permeable for small molecules, and allows for a massive influx of AGAs^22^. However, how sub-lethal intracellular AGA concentrations and the associated mild increase in mistranslation cause damage of membrane proteins is unknown. At bactericidal concentrations, proteotoxic stress induces the heat shock response^13,23^, aggregation of specific sub-proteomes, including membrane and metabolic proteins^24^, protein oxidation and carbonylation^25^, and inclusion body formation^24^. Further downstream effects of AGA treatment are dramatic changes of the cell metabolism, oxidative stress, DNA damage, and ultimately cell death^26–28^. In contrast, sub-lethal AGA concentrations may even be beneficial for bacteria, as they help cells to adapt to stress conditions, change to a more drug-resistant lifestyle (e.g., biofilm formation), or acquire antibiotics resistance^29–33^.

The majority of AGAs affect both decoding and translocation steps of translation. The efficacy of AGAs is often attributed to their ability to induce misreading, rather than to their effect on translocation. In fact, Str, which is bactericidal and induces misreading, has no effect on translocation. Vice versa, spectinomycin (Spc), an AGA that causes a translocation defect without inducing misreading, is bacteriostatic. One potential exception is apramycin (Apr) which is bactericidal, although recent studies suggested that it does not induce misreading^7,34^. This is important, because Apr is less ototoxic than other AGAs in model organisms, presumably because its antibacterial and anti-mitoribosomal activities are uncoupled^7^. Moreover, due to its unique structure, Apr is not susceptible to many prevalent resistance mechanisms and is thus a promising drug for the treatment of multiresistant bacteria^35–40^. Somewhat paradoxically, the bactericidal effect of AGAs is lost when a translation is blocked by bacteriostatic antibiotics, such as chloramphenicol^41^.

Here we used quantitative mass spectrometry to correlate the AGA-dependent cell growth inhibition, miscoding burden, and stress responses, and to obtain insights into the bactericidal effects of AGAs. We found that AGA binding induces not only single errors, but clusters of errors with two, three or four amino acid substitutions located close to each other in the protein sequence. We characterize this type of misreading events for a variety of AGAs and show that their prevalence depends not only on the misreading propensity of a given AGA but also on its ability to inhibit translocation. We also show that Apr, an AGA which is thought not to cause misreading, induces frequent translation errors. These results reveal an additional, unexpected aspect of AGA action in bacteria, suggest how antibiotics cause proteotoxic stress, and provide a simple explanation as to why some misreading-inducing antibiotics are bactericidal.

## Results

### AGA-induced missense errors and cellular fitness

**Fig. 1:**
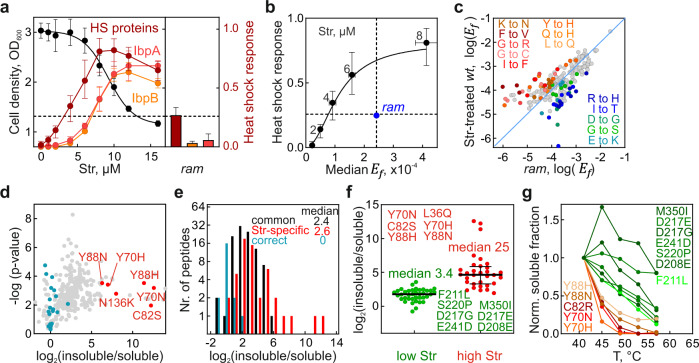
Fitness, stress response, and miscoding of Str-treated *wt* and non-treated error-prone *ram* cells. **a** Str-induced growth inhibition and heat-shock (HS) response with *E. coli wt* cells (left panel) and analysis of the HS response in error-prone *ram* cells without AGA (right panel). Cell density at OD_600_ (left Y-axis; shown are means ± standard deviation (SD) of three biological replicates (*n* = 3)). Proteome changes were quantified by label-free quantification (LFQ). Co-regulated proteins were identified by hierarchical clustering (Supplementary Fig. 1a–e). LFQ values were normalized to interval (right Y-axis). IbpA and IbpB were also plotted individually to show their delayed induction. Shown are medians ± interquartile ranges of three biological replicates (*n* = 3). The dashed line indicates the Str concentration at which the heat shock response is similar to *ram* cells. **b** Correlation between misreading and proteotoxic stress response for Str-treated *wt* cells (black) and untreated *ram* cells (blue). Error frequencies are means ± SEM of the medians of three biological replicates (*n* = 3). HS are median ± interquartile ranges. **c** Misreading profiles of *wt* Str-treated and *ram* cells (6 µM Str; Supplementary Fig. 1g), with Str-specific amino acid substitutions shown in warm colors; amino acid substitutions specific for *ram* strain are in cold colors. Shown are the means of three technical replicates (*n* = 3). **d** Volcano plot visualizing enrichment of amino acid substitutions in aggregates. Distribution between insoluble/soluble fractions of substitutions (gray) was normalized by the median of the correct peptides (turquoise). Selected substitutions enriched in aggregates are highlighted red. Shown are means of three technical replicates (*n* = 3). **e** Aggregation propensity of Str-specific amino acid substitutions. Categories of Str-specific and common errors are derived from Supplementary Fig. 1g (8 µM Str), data for the propensity to be enriched in aggregates from Fig. 1d. **f** Aggregation propensity of proteins with errors induced at low and high Str concentrations. Categories are from Supplementary Fig. 2a, data from Fig. 1d. Median ± interquartile ranges are shown. Unpaired, one-tailed *t* test: *p* value: 1.4 x 10^–8^. **g** Effect of errors on in-vitro thermostability of EF-Tu. Shown are means of three technical replicates (*n* = 3). See also Supplementary Figs. 1, 2, and Source data file.

We first asked what causes the strong detrimental effect of AGAs on bacterial cell viability. To distinguish the AGA-specific response from general stress effects caused by errors, we compared the error load and fitness of *Escherichia coli* cells after AGA treatment with those of untreated *ram* (ribosomal ambiguity) *E. coli* cells that are error-prone due to a C-terminal truncation in ribosomal protein S4^16^. *Ram* cells are viable and have near-*wt* growth rates with only ~30% longer doubling time. For the initial experiments, we used Str, because it does not impede translation^42^. Likewise, the translation rate in *ram* cells is not affected^16,43^, which allows us to attribute potential fitness defects to changes in error levels. Cell viability was estimated using changes in cell density. Proteotoxic stress was assessed by monitoring proteome responses and translational fidelity was determined by data-dependent and targeted mass spectrometry. Hierarchical clustering shows that Str treatment induces the heat-shock response (Supplementary Fig. 1a–e), in agreement with previous reports^13,23^. The upregulation of heat-shock proteins, such as chaperones (e.g., GroEL or DnaK) and proteases (e.g., Lon) (Fig. 1a), is indicative of protein misfolding^44–46^. The small chaperones IbpA and IbpB, which guide unfolded proteins into protein aggregates for their subsequent disaggregation^47^, are induced at higher Str concentrations compared to other heat-shock proteins, and their accumulation coincides with the onset of growth inhibition. Also, *ram* cells have increased levels of heat-shock proteins, compared to the untreated *wt* cells, but the response is mild and comparable to that in *wt* cells treated with Str at sub-lethal concentrations, about 2–4 µM (Fig. 1a; dashed line).

To determine the error frequency (*E*_*f*_) in the same samples, we analyzed translation errors by data-dependent acquisition (DDA) mass spectrometry. We first searched for errors in peptides from elongation factor Tu (EF-Tu), which was chosen because it is constitutively expressed and highly abundant in the *E. coli* lysate, thus allowing us to detect rare misreading events^10^. We quantified peptides that carry single amino acid substitutions and calculated *E*_*f*_ as a ratio of an incorrect peptide to the corresponding correct parental peptide (Supplementary Fig. 1g). As expected, there is a correlation between Str-induced translation errors and the stress response (Fig. 1b). At 2–4 µM Str, *E*_*f*_ in the Str-treated cells is lower than in the *ram* cells, whereas the heat-shock response is similar. At 6–8 µM Str, the error load becomes similar, but at a cost of a much stronger heat-shock response and lower fitness in the Str-treated cells. Notably, some *ram* strains have even higher error loads than the one used in our study^16^. Thus, translation errors per se do not explain the strong stress response and growth inhibition of Str-treated cells.

We next asked why similar apparent error loads that are relatively harmless for *ram* cells are proteotoxic at sub-lethal Str concentrations in *wt* cells. To some extent, this can be a result of the adaptation of *ram* cells to high miscoding levels, which we cannot exclude (Supplementary Fig. 1f). Alternatively, the differences might be attributed to the type of errors, e.g., some types of amino acid substitutions may result in preferential misfolding and aggregation, whereas others may be neutral. Indeed, certain types of amino acid substitutions (e.g., Y → H, G → C, I → F, G → R) are more abundant in Str-treated cells, whereas *ram* cells have more errors involving G-U mismatches in the codon-anticodon complex, such as R → H or E → K substitutions (Fig. 1c). To test whether Str-induced errors result in preferential protein aggregation, we analyzed peptides in aggregated vs. soluble EF-Tu fraction and found that the protein in aggregates indeed contains more errors (Fig. 1d). We then classified the errors as either Str-specific, which we define as more than fivefold more abundant in Str-treated than in the *ram* cells, or common errors, and quantified the distribution between soluble and aggregated fractions for each of the two groups. The error enrichment in protein aggregates is similar for Str-specific and common errors (Fig. 1e); hence, Str-specific errors per se do not cause excessive protein aggregation. However, we note that there is a subgroup of Str-specific errors that appear only at high Str concentrations and are preferentially found in aggregates (Fig. 1d–f and Supplementary Fig. 2a). These errors tend to destabilize proteins, as evident from their reduced thermal stability in vitro (Fig. 1g). Moreover, they become abundant concomitantly with the growth arrest and upregulation of IbpA and IbpB, which indicates an overload of the quality control machinery (Supplementary Fig. 2b, c). Thus, while these destabilizing substitutions start to accumulate at conditions where cells lose viability, they cannot account for the proteotoxic effects seen at sub-lethal AGA concentrations. To understand which types of errors cause early stress responses, we then analyzed the Str-induced missense peptides in greater depth and, surprisingly, identified peptides that had more than one amino acid substitution in the sequence. As such error clusters have not been described before and their appearance might explain the enhanced proteotoxicity of AGAs, in the following we focus our analysis on this particular type of AGA-induced errors.

### Error clusters as a hallmark of AGA action

**Fig. 2:**
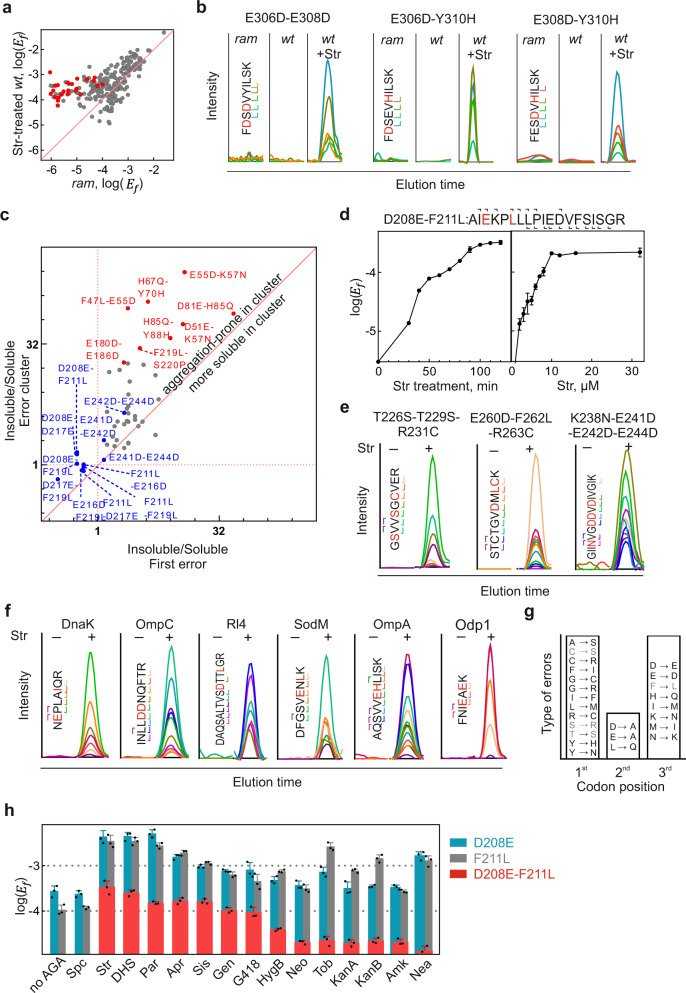
Error clusters as a hallmark of AGA action. **a** EF-Tu-derived peptides with one (gray) or two (red) errors in Str-treated *wt* cells and in untreated *ram* cells. Shown are means of three technical replicates (*n* = 3). **b** Error clusters induced by Str are absent in the *ram* strain. Str concentration (4 µM) was chosen to achieve the same *E*_*f*_ in *wt* and *ram* cells. Peptides with error clusters were detected by QRAS and SRM (rdotp = 1, fragment ions color coded). **c** Enrichment of error clusters in aggregates. After treating cells with Str (8 µM), EF-Tu was isolated from the soluble cell fraction and the insoluble aggregates and peptides analyzed by DDA. Shown are the means of three technical replicates (*n* = 3). **d** Time course (left panel) and concentration dependence (right panel) of Str treatment for D208E-F211L error cluster detected by PRM. Shown are means ± SD of three technical replicates (*n* = 3). **e** Str-induced error clusters with three and four substitutions. Target peptides were enriched by QRAS and detected by PRM. **f** Examples of PRM validation for error clusters in different proteins induced by Str (8 µM). **g** Types of misreading events in error clusters. Errors with ambiguous classification of the misreading position are shown in gray; in cases where misreading can arise from mismatches at different positions, the errors were classified as originating from the 3^rd^ > 1^st^ > 2^nd^ position mismatch, according to their response to AGA treatment^9–11^. **h** Effect of different AGAs. The error cluster D208E-F211L in EF-Tu detected by PRM upon treatment with spectinomycin (Spc), streptomycin (Str), dihydrostreptomycin (DHS), paromomycin (Par), apramycin (Apr), sisomycin (Sis), gentamycin (Gen), geneticin (G418), hygromycinB (HygB), neomycin (Neo), tobramycin (Tob), kanamycin A (KanA), kanamycin B (KanB), amikacin (Amk), or neamine (Nea) at concentrations that gave rise to maximum misreading (‘Methods’). Shown are means ± SD of three technical replicates (*n* = 3). See also Supplementary Figs. 1–3 and Source data file.

To identify further peptides with two or more amino acid substitutions, we first searched the Str-treated samples for peptides with more than one amino acid substitution by DDA (Fig. 2a and Supplementary Fig. 3a) and validated their existence in the lysate of the *E. coli* reference strain MG1655 by Parallel Reaction Monitoring (PRM) using synthetic reference peptides (Supplementary Fig. 4a). Next, we predicted potential clusters based on the experimentally measured frequencies of single amino acid substitutions (‘Methods’). Guided by these predictions, we enriched the expected candidate peptides by QRAS, a method developed for the Quantification of Rare Amino acid Substitutions^10^. QRAS relies on the chromatographic enrichment of missense peptides using synthetic reference peptides as standards, resulting in the improvement of the signal-to-noise ratio and high confidence identifications. We found a large number of peptides with two amino acid substitutions, which were either adjacent or interspersed by several correct amino acids (Supplementary Fig. 4b). Error clusters appear only in Str-treated samples and are absent in *ram* cells (Fig. 2b). Error clusters are enriched in aggregates, but the effect depends on the type of errors (Fig. 2c). While in many cases both single errors and their combinations are well tolerated (e.g., D208E and F211L, Fig. 1f, g, and D208E-F211L, Fig. 2c (blue symbols)), in other cases the increased error load leads to enhanced aggregation (Fig. 2c, red symbols).

Although the prevalence of error clusters depends on Str concentration and treatment duration, they are clearly detectable already after short incubation times and at low, sub-lethal Str concentrations, which shows that their occurrence is not related to cell death (Fig. 2d). Moreover, we also found error clusters with three or four amino acid substitutions in one peptide sequence (Fig. 2e and Supplementary Fig. 4b). To ensure that error clusters are prevalent in the proteome, we also analyzed different full-length proteins in the *E. coli* lysate (Fig. 2f and Supplementary Fig. 3a). Overall, we validated 96 error clusters with errors originating from mismatches in the first, second, or third position of the codon–anticodon complex (Fig. 2g, Source data file).

To study whether error cluster formation is caused by AGAs other than Str, we used AGAs from different structural classes (Fig. 2h). All bactericidal AGAs tested give rise to error clusters; spectinomycin (Spc), which is bacteriostatic, does not induce error clusters. Surprisingly, also Apr, which was reported not to cause significant misreading^7,34^, induces single errors and error clusters. The frequency of error clusters ($E_f^{\mathrm{cluster}}$) is in the same range as *E*_*f*_ for most single amino acid substitutions. Overall, this shows that the appearance of error clusters is a hallmark of the action of bactericidal AGAs.

### Apr effect on misreading and translation

**Fig. 3:**
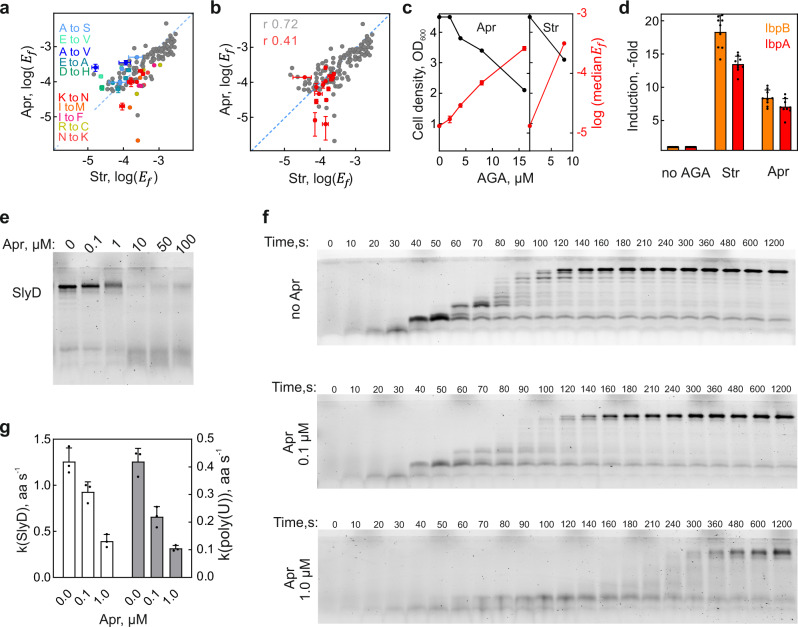
Apramycin induces proteotoxic misreading and inhibits translocation. **a** Single amino acid substitutions induced by Apr and Str. Errors induced predominantly by Apr (16 µM) are shown in cold colors; those induced by Str (8 µM) in warm colors. Shown are means ± SD of three technical replicates (*n* = 3). **b** Error clusters in Apr-treated cells (red) compared to single errors (gray). Shown are means ± SD of three technical replicates (*n* = 3). We note a better correlation for single errors (Pearson coefficient 0.72) than for error clusters (0.41), suggesting that the mechanisms that govern cluster formation are not identical to those for single errors and depend on the AGA used. **c** Growth defect and error load at increasing Apr concentrations. At growth-inhibitory concentration of Apr the median error frequency approaches that after Str treatment. Data from Supplementary Fig. 5b; Shown are means ± SD of three technical replicates (*n* = 3). **d** Induction of the heat shock response. The chaperones IbpA/IbpB are significantly induced upon Apr (16 µM) and Str (8 µM) treatment as quantified by SRM. Shown are means ± SD of three technical replicates (*n* = 3) calculated taking into account their individual peptide-based stoichiometries. **e** Products of the in-vitro translation of SlyD mRNA. Translation products are separated by SDS PAGE and detected using the fluorescence of the N-terminal BODIPY-Met. **f** Time courses of in-vitro translation of SlyD mRNA at different Apr concentrations. Translation products were detected as in (**e**). **g** Rate of in-vitro translation (aa/s) at different Apr concentrations for SlyD mRNA (white bars) and poly(U) mRNA (gray bars). Shown are means ± SD of three technical replicates (*n* = 3). See also Supplementary Fig. 5 and Source data file.

The surprising finding that Apr induces errors prompted us to study the effects of Apr on misreading and translation in more detail. We validated that all Apr batches purchased from different vendors induced significant misreading (Supplementary Fig. 5a). Most errors that are induced by Str are induced by Apr as well, including the error clusters (Fig. 3a, b and Supplementary Fig. 5b). Str and Apr induce very similar error loads and proteotoxic stress response at concentrations that inhibit growth (Fig. 3c, d). To better understand why recent in-vitro studies came to a different conclusion^7,34^, we tested how Apr affects protein synthesis in a fully reconstituted translation system. As mRNAs we used a natural mRNA coding for the model protein SlyD and a homopolymeric mRNA (poly(U)) coding for polyphenylalanine. At Apr concentrations of 0.1–1 µM translation is impaired (Fig. 3e–g). For SlyD, both the end level (Fig. 3e and Supplementary Fig. 5c) and the rate (Fig. 3f, g and Supplementary Fig. 5c) of translation are affected. The very low translation efficiency, which was also noted in the previous reports^7,34^, might have masked the occurrence of the misreading events in those experiments. Apr also reduces the rate of poly(U) translation with Phe-tRNA^Phe^ (Fig. 3g and Supplementary Fig. 5d), i.e., at conditions where no misreading can occur due to the lack of near-cognate tRNA, supporting the suggested effect of Apr on translocation (the rate of peptide bond formation is not affected by the AGA)^34^. In summary, Apr, as other bactericidal AGAs, increases the elongation time and impairs translation fidelity by inducing single amino acid substitutions and error clusters in proteins.

### Probability of making successive errors

**Fig. 4:**
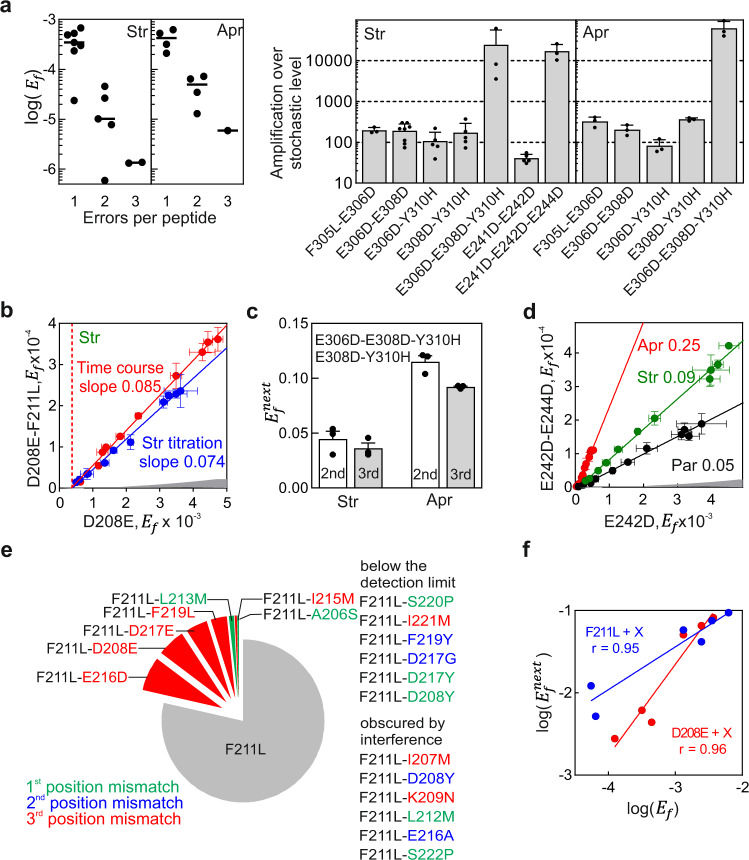
Frequencies of consecutive errors. **a** Absolute quantification of error clusters by QRAS. Left panels: *E*_*f*_ of one, two, or three amino acid substitutions after AGA treatment. Dash is a median value shown as a visual guide. Right panels: Error amplification is calculated as $\frac{{E_f^{\mathrm{cluster}}}}{{E_f^{\mathrm{1st}} \times E_f^{\mathrm{2nd}}}}$, where $E_f^{\mathrm{1st}} \times E_f^{\mathrm{2nd}}$ represents the expected stochastic occurrence of error cluster. Shown are means ± SD of at least three independent biological replicates (*n* = 3). **b** Correlation between $E_f^{\mathrm{1st}}$ and $E_f^{\mathrm{cluster}}$. Peptides were quantified by PRM and LFQ assuming identical ionization and fragmentation properties. Shown are means ± SD of three technical replicates (*n* = 3). **c** $E_f^{\mathrm{next}}$ for the 2^nd^ and 3^rd^ errors in the cluster. $E_f^{\mathrm{next}}$ is calculated as $\frac{{E_f^{{\mathrm{E}}308{\mathrm{D}} - {\mathrm{Y}}310{\mathrm{H}}}}}{{E_f^{\mathrm{E}308\mathrm{D}}}}{\mathrm{and}}\frac{{E_f^{{\mathrm{E}}306{\mathrm{D}} - {\mathrm{E}}308{\mathrm{D}} - {\mathrm{Y}}310{\mathrm{H}}}}}{{E_f^{{\mathrm{E}}306{\mathrm{D}} - {\mathrm{E}}308\mathrm{D}}}}$. Shown are means ± SD of three independent biological replicates (*n* = 3). **d** Example of $E_f^{\mathrm{next}}$ for the E242D–E244D cluster induced by different AGAs. Gray shaded area represents the expected stochastic error cluster frequency for the two positions. Shown are means ± SD of three technical replicates (*n* = 3). **e** Misreading preferences in error clusters. Str-induced error clusters were quantified relative to their parental peptide with a single substitution (F211L) by PRM and LFQ. **f** Correlation in occurrence of an error as a single substitution (*E*_*f*_) or within an error cluster $(E_f^{\mathrm{next}})$. Data for the probability of single errors is derived from Supplementary Fig. 1g; the probability to occur in error cluster is based on Fig. 4e and Supplementary Fig. 6. Pearson correlation coefficients as insets. See also Supplementary Fig. 6 and Source data file.

Given that all bactericidal AGAs including Apr induce error clusters, we next asked whether consecutive errors in the same peptide can be explained by their stochastic occurrence. For errors that occur stochastically and are independent of each other, the expected $E_f^{\mathrm{cluster}}$ is a product of individual *E*_*f*_ values for each position, which would be <10^–6^–10^–5^ in most cases. In contrast, the observed error clusters can be almost as frequent as most single amino acid substitutions, indicating that consecutive misreading events occur non-randomly (Figs. 2a, 3b). To estimate how non-random these events are, we determined by QRAS the $E_f^{\mathrm{cluster}}$values for clusters with two or three errors, as well as the *E*_*f*_ values for the respective single amino acid substitutions, and compared the experimental and the estimated stochastic values (Fig. 4a and Supplementary Fig. 4c). The measured error frequency for clusters is up to 60,000-fold higher than that predicted for a combination of random misreading events at the same positions, largely independent of the detection method (SRM, PRM vs. DDA) or the protein (EF-Tu or other model proteins, Supplementary Fig. 3b). Due to the vectorial nature of translation, the appearance of the first error is independent of all subsequent misreading events, which implies that after the first error is made, all subsequent errors are much more frequent than expected. Analysis of the Str data shows that the $E_f^{\mathrm{cluster}}$ value depends linearly on the *E*_*f*_ value of the first misreading event ($E_f^{\mathrm{1st}}$) and the slope of the plot is independent of the type of experiment (Fig. 4b). The slope of 0.08 in the example of Fig. 4b provides an estimate for the error frequency of the second misreading event, which we denote as $E_f^{\mathrm{next}};E_f^{\mathrm{1st}} \times E_f^{\mathrm{next}} = E_f^{\mathrm{cluster}}$. For a given type of error clusters, $E_f^{\mathrm{next}}$ values are largely independent of the protein tested and the position of the cluster in the protein sequence (Supplementary Fig. 3c). $E_f^{\mathrm{next}}$ for the second and third substitutions are very similar (Fig. 4c). The propensity to make consecutive errors depends on the AGA, e.g., for a given peptide the $E_f^{\mathrm{next}}$ is 0.09 and 0.05 for Par and Str, respectively, but is much higher, 0.25, for Apr (Fig. 4d). The $E_f^{\mathrm{next}}$ values provide a lower-limit estimate for the intrinsic error frequency of decoding on those ribosomes where an AGA is bound, i.e., at conditions where misreading is independent of the influx or the intracellular concentration of an AGA.

AGAs preferentially induce misreading in the third codon position^9–11^. To test whether this tendency applies to consecutive errors, we inspected misreading preferences in clusters for a fixed amino acid substitution, such as F211L or D208E, both likely induced by third-position misreading. Indeed, the majority of misreading events in the clusters can be explained by third-position misreading, whereas substitutions due to the first and second position mismatches are clearly less abundant (Fig. 4e and Supplementary Fig. 6a, b). If a particular error is frequent as a single error, it is also frequent as an error in the cluster (Fig. 4f), suggesting that the preference for a particular type of misreading is conserved in error clusters.

### Distance dependence of successive errors

**Fig. 5:**
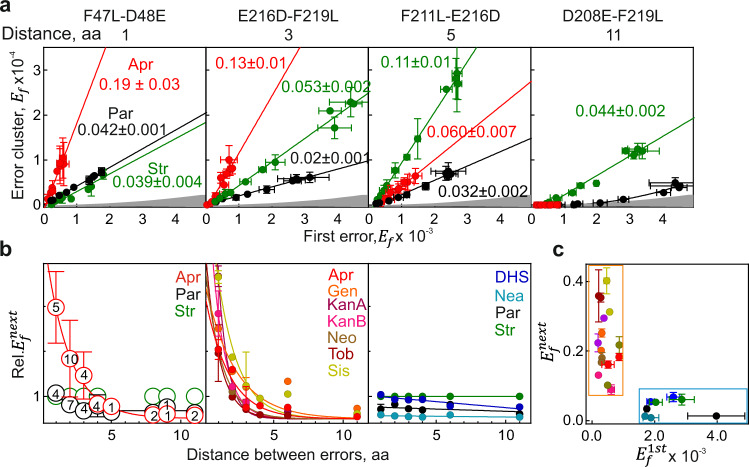
Distance dependence of successive errors. **a** Correlation between $E_f^{\mathrm{1st}}$ and $E_f^{\mathrm{cluster}}$ for different AGAs and clusters. Slopes of the linear plots yield the $E_f^{\mathrm{next}}$values. Shown are means ± SD of three technical replicates (*n* = 3). Expected stochastic level of error clusters is indicated as gray area. **b** Distance dependence of error cluster formation. Left panel: Distance dependence $E_f^{\mathrm{next}}$ for Apr, Par, and Str. Data from Figs. 4a, d, 5a, and Supplementary Figs. 7a, 8. The probability for error cluster formation is normalized to that of Str value, which has very little distance dependence and is set to 1 (‘Methods’). The number of clusters is indicated inside the symbols. When more than one cluster per distance was inspected, mean values ± SD are shown. Middle and right panel: Distance dependence of error cluster formation for different AGA based on PRM measurements. For single clusters, a mean of technical replicates is shown (*n* = 3). If two or more clusters were considered for a given distance, the symbols represent a mean ± SD. **c** Correlation between $E_f^{\mathrm{1st}}$ and $E_f^{\mathrm{next}}$ for different AGAs. Data and color code are from (**b**). Only clusters with adjacent substitutions are considered (F47L-D48E; E241D-E242D). Shown are means ± SD of three technical replicates (*n* = 3). See also Fig. 4a, d, Supplementary Figs. 7, 8, and Source data file.

Finally, we determined the frequency of successive errors $(E_f^{\mathrm{next}})$ in different error clusters for different AGAs (Figs. 4d, 5a, and Supplementary Fig. 7a). $E_f^{\mathrm{next}}$ with Str or Par shows only slight variation, $E_f^{\mathrm{next}}$ = 0.04–0.18 and 0.02–0.09, respectively. However, with Apr the $E_f^{\mathrm{next}}$ value is highly variable, as in some cases no error clusters are detected, whereas in other cases $E_f^{\mathrm{next}}$reaches 0.25. We then noticed that with Str the frequency of the successive error is almost independent of the distance between the incorrect amino acids in a peptide. In contrast, with Apr the probability of the next misreading event decreases dramatically with distance; with Par the dependence is very weak and the $E_f^{\mathrm{cluster}}$ value for the two distant misreading events is close to the stochastic one (Fig. 5a, b, left panel). We then analyzed the distance dependence of error cluster formation for other AGAs based on a subset of error clusters. With several AGAs (e.g., Apr, sisomycin (Sis), Neo, Tob, Gen, or KanA and KanB), $E_f^{\mathrm{next}}$ is very high for closely spaced errors (e.g., 0.4 for Sis and E241D–E242D) (Fig. 5c), but strongly decreases with distance (Fig. 5b, middle panel). The most likely explanation for the distance dependence is that the AGA dissociates from the ribosome within several rounds of elongation. Exponential fitting of the distance dependencies suggests that the probability to continue translation with the AGA still bound is about 0.3–0.7 for the cases shown in Fig. 5, middle panel, suggesting that the rates of translation and AGA drop-off are comparable. In contrast, with other AGAs, such as Par, Str, dihydrostreptomycin (DHS), and neamine (Nea), there is very little distance dependence (Fig. 5b, right panel). A replot of $E_f^{\mathrm{next}}$vs. $E_f^{\mathrm{1st}}$ (Fig. 5c) suggests that there are two groups of AGAs, those that induce moderate misreading for single amino acid substitutions, but once bound—induce frequent local consecutive errors (Apr, Gen, Kan, Sis, Tob), and those that have a strong misreading effect on a single position, but are less efficient in inducing error clusters (DHS, Nea, Par, Str). These data show that, although all AGAs that induce misreading can induce clusters of missense errors, the prevalence of such errors and the distance between consecutive errors in a cluster depends on the nature of the AGA.

## Discussion

The analysis of AGA-induced translation errors in cellular proteins by mass spectrometry reveals the existence of prevalent clusters of translation errors, which is a distinct class of misreading events in vivo and likely explains why most AGAs are bactericidal. The comparison of AGA-induced errors in *wt E. coli* cells with those in an error-prone *ram* strain shows that the error frequency or the type of errors alone do not account for the strong stress response and fitness loss upon mild AGA treatment. Although single errors induced at low AGA concentrations do not cause excessive protein aggregation, heat-shock proteins are upregulated to a much higher extent than in the *ram* strain, which has a much higher error level, but shows only a moderate heat-shock response. At high AGA concentrations, the high error load results in the accumulation of destabilized protein variants, formation of protein aggregates, upregulation of the chaperones IbpA and IbpB, and a concomitant loss of cell viability. This “kiss-of-death” response is much more dramatic than a mild heat-shock response detected in the *ram* cells at a comparable error load. These observations show that AGA causes specific defects that induce an early heat-shock response and compromise cell fitness. Error clusters with multiple translation errors appear early upon AGA treatment and at low AGA concentrations, but are not found in the *ram* proteome. Regardless of the frequency of the first error in the string, all subsequent errors are much more abundant than if they were stochastic. Furthermore, the probabilities of the second and third errors in a cluster are very similar. These two observations strongly suggest that the AGA stays bound to the ribosome over a number of elongation cycles, thereby inducing consecutive errors. This increases the misreading potential at low AGA concentrations by orders of magnitude and makes all the downstream errors in a cluster independent of the AGA concentration. Error clusters can thus constitute the predominant source of proteotoxic effects, in particular at low AGA concentrations or in the initial phase of AGA treatment where the median error frequency for single errors is low. Moreover, our data indicate that proteins with error clusters are somewhat more prone to aggregation than those with single errors. While the effect of multiple amino acid substitutions on stability is often additive, the combinatorial effect on cellular fitness is often stronger, which is known as negative epistasis^48^. The threshold model explains this behavior assuming that first mutations are buffered by the excess of stability, whereas subsequent mutations reduce the stability beyond the allowed stability margin^48^.

The error clusters described here are fundamentally different from the spontaneous consecutive misreading events that can occur in the absence of the AGA and cause premature translation termination^49^. Termination produces incomplete proteins that are likely to be degraded by the cellular quality control machinery. Peptides with adjacent amino acid substitutions at the C terminus that likely represent the premature termination products were recently detected by mass spectrometry upon translation of model constructs^50^. In contrast to such spontaneous misreading events, AGA-induced error clusters are found in full-length proteins, although we cannot exclude that a fraction of nascent peptides with two adjacent errors is degraded via the termination editing pathway. Furthermore, AGA-induced translation errors are not necessarily sequential; rather, individual misreading events can be separated by as much as 13 correct elongation cycles (Supplementary Fig. 6b and Source data file). The AGA-induced error clusters are induced by all bactericidal AGAs tested, underscoring the importance of this phenomenon for understanding the mode of AGA action.

We provide the first characterization of error clusters induced by different AGAs. In general, they emerge already at low AGA concentrations and early after the beginning of the AGA treatment. Although the probability to make the first error varies with the nature of the AGA, its concentration, and the time of treatment, the probability of a subsequent error depends only on the nature of the AGA. The preference for a particular type of misreading event (e.g., a third codon position mismatch) is common for the first and subsequent substitutions. These observations suggest that the propensity of the second and subsequent errors depends on the error-prone conformation of the ribosome and the residence time of the particular AGA on the ribosome once it is bound.

**Fig. 6:**
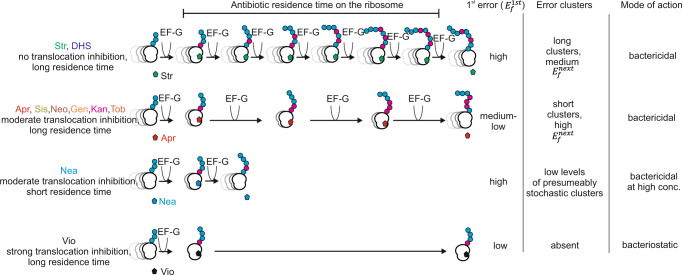
Model of AGA-induced error cluster formation. The efficacy of an AGA in inducing proteotoxic error clusters depends on its ability to bind and induce the 1^st^ error, its residence time on the ribosome, and the inhibitory effect on translocation. Str, which does not affect translocation, and its derivative DHS, induce frequent single errors and remain bound to the ribosome for many elongation cycles, resulting in formation of long error clusters. AGAs that have a moderate inhibitory effect on translocation induce short clusters, because they tend to dissociate after only a few slow translation rounds. Neamine has a high misreading potential, but dissociates rapidly from the ribosome, resulting in low levels of presumably stochastic error clusters which are thus not distance dependence. Viomycin stops translocation which terminates translation by ribosomes that have the antibiotic bound and hence prevents formation of the error clusters.

Based on their efficacy in inducing single and multiple errors, bactericidal AGAs can be subdivided into two groups, (I) those that are potent misreading agents for single substitutions, but have a moderate efficacy in inducing error clusters, and (II) those that have a moderate effect on single errors, but induce very strong misreading in a narrowly spaced cluster sequence. One characteristic example of the group I AGA is Str, which is a potent misreading agent that does not affect translocation. Analysis of the error clusters suggests that Str remains bound to the ribosome for at least ten elongation cycles, thereby inducing long error clusters with a relatively moderate error frequency for each consecutive error (Fig. 6). The error frequency of consecutive errors in the cluster reaches 0.18, which is close to the maximum misreading value of 0.25 measured in vitro at saturating Str concentration^51^. Nea, which also belongs to group I, is inefficient in inducing error clusters probably because it inhibits translocation and dissociates rapidly from the ribosome^2,52^ (Fig. 6). The low affinity of Nea to the ribosome and the resulting inability to promote error clusters may explain its high MIC (64 µg/ml Nea vs 1 µg/ml Neo)^53^. Par induces high levels of single errors, but has a relatively low propensity to induce error clusters, and the occurrence of long-distance clusters is close to the expected stochastic level.

The majority of AGAs belong to group II. They induce a medium-low error frequency for the first position in a cluster, but the frequency of the next error is very high, in particular for KanA and Sis, reaching 0.36 and 0.40, respectively, which means that when the antibiotic is bound, up to 36% or 40% of the ribosomes can misread the next codon. These AGAs affect translocation in addition to misreading^2,34^. The rate of translocation on the ribosomes that have an AGA bound is comparable to that of the AGA dissociation, which results in a gradual loss of the AGA with each elongation cycle and explains the strong distance dependence of the error frequency in the cluster (Fig. 6). This is consistent with in-vitro single-molecule FRET data showing that AGAs can stay on the ribosome for several elongation cycles^52,54^. Together, these results indicate that the rates of translocation in the presence of AGA and of AGA dissociation, as well as the ribosome conformation induced by AGA binding, define the prevalence and the size of error clusters. Apr induces pronounced error hot spots, whereas Str introduces milder, but far longer error clusters, some of which are likely to supersede the length of average tryptic peptides.

The notion that misreading alone does not explain the bactericidal effects of AGA has important clinical implications. Noteworthy, most of the global protein synthesis inhibitors are bacteriostatic^55^. For example, chloramphenicol and viomycin induce translation errors, but are bacteriostatic^56–59^. This is because after binding the antibiotic, the ribosome that made a mistake stops translation. In fact, peptides with an incorrect C-terminal amino acid were identified by mass spectrometry after chloramphenicol treatment of *E. coli* cells^60^. The appearance of such aberrant protein fragments, which may be removed by the quality control machinery, may be less harmful for the cell than the accumulation of full-length proteins with error clusters. Similarly, chloramphenicol rescues the bactericidal effect of AGAs^41^ probably because the inhibition of translation by chloramphenicol would preclude the AGA-induced formation of error clusters and thus avoid synthesizing aberrant proteins. The balance between effects on decoding and translocation might be species-dependent^61^. Bacteriostatic drugs might become bactericidal in some organisms if the residual translocation speed in the presence of the antibiotics is higher due to alterations in the ribosome structure. For Apr, its strong effect on misreading and translocation, combined with specific features of translation in mitochondria, might explain why Apr is less oto- and nephrotoxic than other AGAs.

In summary, our results suggest that bactericidal AGAs can stay bound to the ribosome for several elongation cycles resulting in a high probability of multiple consecutive misreading events. While the error frequency of single amino acid substitutions depends mostly on the stochastic binding of AGA to the ribosome, subsequent errors are determined (i) by the propensity of the AGA-induced conformation of the ribosome to incorporate near-cognate amino acids and (ii) on the residence time of the AGA on the ribosome compared to the translocation rate. In a sequence context where many codons allow for third position mismatches, consecutive errors result in aberrant proteins whose abundance is much higher than expected from the stochastic combination of individual error frequencies at low AGA concentrations. This potentiation of the misreading effect for consecutive errors in the cluster and the higher likelihood of error clusters to induce aggregation would explain how very low concentrations of AGAs initiate the autocatalytic uptake of the drugs, presumably by inducing the formation of defective membrane proteins. Moreover, the aminoglycoside-induced synthesis of highly aberrant aggregation-prone proteins could explain how bacteria sense proteotoxic stress at low aminoglycoside concentrations to develop drug resistance or communicate with other bacteria in the population^31,62^. Future studies will reveal to which extent and which error clusters lead to the precipitation of specific subproteomes, destabilize the membrane integrity in the initial phase of AGA uptake, or promote mitochondrial proteotoxic stress in eukaryotes. Notably, such errors might contribute to side effects when AGAs are used to suppress premature stop codons in humans. As a distinct class of AGA-induced responses, error clusters are of major importance for the understanding of AGA action and may guide the development of new generations of antimicrobial drugs.

## Methods

For experimental details, MS acquisition parameters, and an error cluster overview, see Source data file. See also the Supplementary Table 1 in the Supplementary Information.

### Chemicals & AQUA peptides

Chemicals were purchased from Merck or Sigma if not stated otherwise. Chemicals used for chromatography were of HPLC/MS grade. Samples were handled in low retention reaction cups (Eppendorf). AQUA peptides for absolute quantification (QRAS) of missense peptides were purchased from Thermo Scientific. For the quantification of correct peptides Ultimate grade AQUA peptides were used (guaranteed concentration error <5%); for missense peptides, QuantPro grade was used (guaranteed concentration error <25%). For secure identification of missense peptides in LFQ, isotope-labeled reference peptides were used from JPT (spikeTidesL).

### Bacterial strains and cell growth

*E. coli* reference strain MG1655 was purchased from the German Collection of Microorganisms and Cell Cultures (Braunschweig, Germany). *E. coli* cells with chromosome-encoded EF-Tu with a C-terminal His-tag were obtained based on the W3110 (K12) strain^10^. Xac (araΔ[lacproAB]ryrA rpoB argEamber and UD 131 (Xac rpsD12)^16^ was kindly provided by Dr. Hani Zaher (Washington University, MO, USA).

*E. coli* cells were grown in LB medium at 37 °C. Unless stated otherwise, 500 ml cultures were grown at 200 rpm to OD_600_ of 0.3 and treated with AGA for 2 h. If not stated otherwise, the Str concentration was 8 µM. AGA concentrations that gave rise to maximum misreading (Figs. 2h, 5b, c) were determined by titrations: Str (8 µM), DHMS (16 µM), Apr (16 µM), HygB (128 µM), Nea (64 µM), Neo (8 µM), Par (8 µM), Gen (2 µM), G418 (2 µM), Sis (8 µM), Tob (4 µM), KanA (16 µM), KanB (8 µM), Amk (8 µM). Spc was used at a concentration of 64 µM, which is the minimal concentration that inhibits cell growth.

### In-vitro translation

Initiation complex with native mRNA (*slyD*) were prepared in buffer A (50 mM HEPES pH 7.5, 70 mM NH_4_Cl, 30 mM KCl, 7 mM MgCl_2_, 2 mM DTT, and 2 mM GTP). Ribosomes (0.5 μM) were incubated with initiation factors (IF1, IF2, and IF3; 2.25 μM each), SlyD mRNA (2 μM), and BodipyFL-[^3^H]Met-tRNA^fMet^ (1 µM) for 45 min at 37 °C. Ternary complex EF-Tu–GTP–aa-tRNA was prepared in buffer A by incubating EF-Tu–GDP (120 μM) with phosphoenolpyruvate (3 mM), and pyruvate kinase (0.05 mg/mL) for 15 min at 37 °C, mixing with total aminoacyl-tRNA (200 μM), and further incubation for 1 min at 37 °C. In-vitro translation was performed in HiFi buffer (50 mM HEPES pH 7.5, 70 mM NH_4_Cl, 30 mM KCl, 3.5 mM MgCl_2_, 1 mM DTT, 0.5 mM spermidine, and 8 mM putrescine)^51^. Initiation complexes (80 nM) were mixed with EF-Tu–GTP–aa-tRNA (100 μM) and EF-G (1 μM), and incubated at 37 °C with or without Apr. Translation products were separated by Tris-Tricine gel electrophoresis^63^. Fluorescent peptides were detected using Starion IR/FLA-9000 scanner (FujiFilm) and quantified using Multi Gauge software. The rate of translation was calculated by dividing the length of SlyD protein by the time of translation obtained from the time courses of SlyD synthesis.

To translate polyU, ribosomes (1.5 μM) were first incubated with polyU mRNA (1 mg/ml) and Ac[^14^C]Phe-tRNA^Phe^ (1.8 µM) on ice for 2 h in buffer B (50 mM HEPES pH 7.5, 70 mM NH_4_Cl, 30 mM KCl, 20 mM MgCl_2_, 2 mM DTT, and 2 mM GTP), which results in binding of AcPhe-tRNA to the P site of the ribosome. EF-Tu–GTP–aa-tRNA was prepared as described above using EF-Tu–GDP (15 μM) and [^14^C]Phe-tRNA^Phe^ (5 μM). In-vitro translation was performed in HiFi buffer (see above). 70S–polyU–AcPhe-tRNA (50 nM) was mixed with EF-Tu–GTP–[^14^C]Phe-tRNA^Phe^ (4.2 μM) and EF-G (1 μM), and incubated at 37 °C with or without Apr. Translation was stopped by addition of 0.5 M KOH. The translation product poly(Phe) was precipitated by 10% trichloroacetic acid, collected by nitrocellulose filtration, and quantified by scintillation counting. The rate of translation was calculated by dividing the average length of poly(Phe) peptide by the time of translation obtained from the time courses using [^14^C] counts.

### EF-Tu purification for peptide analysis

If not state otherwise, EF-Tu has isolated from *E. coli* lysate by SDS PAGE, the respective band excised, and in-gel digested^64^. For quantitative analysis, EF-Tu was purified under denaturing conditions. Cells were opened in buffer C (25 mM HEPES–KOH pH 7.5, 8 M urea, 200 mM KCl, 10 mM MgCl_2_, 5 mM β-mercaptoethanol) by sonification. Affinity purification was carried out using a Protino Ni-IDA column according to manufacturer’s protocol. After elution, the protein was rebuffered in buffer D (100 mM NH_4_HCO_3_, 8 M urea) in an Amicon Ultra 4 concentrator with a 30 kDa cutoff (Merck Millipore) and proteolyzed by in-solution digestion.

For native purification of His-tagged EF-Tu, cells were solubilized in B-PER reagent (Thermo Scientific) supplemented with 200 mM KCl, 3 mM MgCl_2_, Complete Protease Inhibitor (1 tablet per 50 ml, Roche Diagnostics), 30 μM GDP, 5 mM β-mercaptoethanol, and traces of DNase I (Sigma Aldrich). Solubilized cells were sonicated for 1 min and cell debris removed by centrifugation. EF-Tu was purified using Ni-IDA Protino columns (Macherey-Nagel) according to manufacturer’s protocol. EF-Tu was stored in buffer E (50 mM Tris–HCl pH 7.5, 70 mM NH_4_Cl, 30 mM KCl, 7 mM MgCl_2_) at −80 °C.

For native purification of untagged EF-Tu, cells were resuspended and lysed in buffer F (50 mM HEPES–KOH pH 7.5, 50 mM KCl, 10 mM MgCl_2_, 5 mM β-mercaptoethanol, containing Complete Protease Inhibitor (1 tablet per 50 ml, Roche Diagnostics) and traces of DNase I (Sigma Aldrich)). Cells were lysed using the EmulsiFlex C3 (Avestin). Cell debris was removed by centrifugation. Lysate was loaded on a HighTrap Q HP anion exchange column (5 ml, GE Healthcare) and eluted using a salt gradient in buffer G (5–400 mM KCl in 25 mM HEPES–KOH pH 7.5, 3 mM MgCl_2_, 5 mM β-mercaptoethanol, 30 μM GDP). EF-Tu-containing fractions were applied to two sequential purification steps by SEC (HiLoad 26/60 Superdex 75, prep grade, GE Healthcare). EF-Tu-containing fractions were pooled, re-buffered into buffer H (50 mM HEPES–KOH pH 7.5, 50 mM KCl, 10 mM MgCl_2_), concentrated, and stored at −80 °C.

### In-vitro thermostability of EF-Tu

To access the impact of errors on thermostability EF-Tu from cells treated with Str (8 µM) was stepwise denatured by heating. The temperature was gradually increased (4 °C steps starting at 37 °C, 5 min incubation each). At each temperature, aggregates were removed by centrifugation and the soluble protein fraction was analyzed by DDA-MS. The signal of peptides with individual amino acid substitutions was normalized to the signal of the corresponding correct parental peptides. The signal for 41 °C was set to 1.

### Proteolysis

For the in-solution proteolysis of EF-Tu purified under denaturing conditions, a modified FASP (filter-aided sample preparation) procedure^65^ was applied. To reduce disulfide bonds, His-tagged EF-Tu purified under denaturing conditions was re-buffered to buffer I (100 mM NH_4_HCO_3_, 8 M urea, 20 mM DTT) using a 30 kDa concentrator at room temperature. After the volume was reduced tenfold, a tenfold excess of buffer J (100 mM NH_4_HCO_3_, 8 M urea, 100 mM iodoacetamide) was added to alkylate thiol groups and the protein was further concentrated. After 30 min of alkylation, EF-Tu was rebuffered to buffer D. The urea concentration was lowered to 1–2 M by adding 100 mM NH_4_HCO_3_. Trypsin (Trypsin Gold, Promega) was added to the protein at a substrate:protease ratio of ~200:1, and EF-Tu was proteolyzed for 16 h at room temperature. Tryptic peptides were eluted by centrifugation through the 30 kDa cutoff filter and the concentrator was washed with H_2_O. Samples from both elution steps were pooled.

For proteolysis of EF-Tu prepared under native conditions, 3–100 nmol of EF-Tu was precipitated overnight with five volumes of ice-cold acetone at −20 °C. Protein was collected by centrifugation, washed with ice-cold 80% ethanol, and the pellet dried. EF-Tu was resuspended in 1% RapiGest (Waters) in 25 mM NH_4_HCO_3_ and incubated for 10 min at 37 °C. Disulfide bonds were reduced by addition of 20 mM DTT (in 25 mM NH_4_HCO_3_) in two incubation steps, at 60 °C for 10 min, and at 37 °C for 20 min. Alkylation of thiols was performed in 30 mM iodoacetamide (in 25 mM NH_4_HCO_3_) and incubating the sample at RT for 30 min in the dark. RapiGest (Waters) in the sample was diluted to 0.1% with 25 mM NH_4_HCO_3_. Trypsin (1 μg/μl) (Trypsin Gold, Promega) was added to the sample (final concentration 0.01 μg/μl) and EF-Tu proteolyzed overnight at 37 °C. In-gel proteolysis was carried out according to the standard protocol^64^.

### Proteome analysis

Cells (1 OD_600_) were opened in SDS loading buffer. Proteins (corresponding to 0.1 OD_600_ of cells) were desalted by a 10% Mini-PROTEAN TGX SDS PAGE (BIORAD). Proteins were stained with Coomassie and in-gel proteolyzed with trypsin.

Samples were analyzed on an Ultimate 3000 RSLC system coupled to a QExactive HF Hybrid Quadrupole-Orbitrap mass spectrometer. Tryptic peptides were loaded on a C18 precolumn (2.5 cm length, 150 µm inner diameter, Reprosil-Pur 120 Å, 5 µm (Dr MaischGmbH, Germany)). Bound peptides were eluted and separated on a C18 capillary column (31 cm, 75 µm ID, Reprosil-Pur C18-AQ 120 Å C18, 1.9 µm) at a flow rate of 300 nl/min, with a 45 min linear gradient from 5 to 45% acetonitrile (ACN) in 0.1% formic acid. Analysis was performed in positive ion mode using a Top 30 DDA method. MS survey spectra were acquired at a resolution of 60000 FWHM (Full Width at Half Maximum) in the range of 350–1600 *m*/*z*. Automatic gain control (AGC) target values and maximum injection times for MS runs were 1e^6^ and 50 ms. Precursors with charge states *z* = 2–5 above the intensity threshold of 1.2 e^4^ were selected at an isolation width of 1.6 m/z for fragmentation by higher energy collision dissociation (HCD) with a normalized collision energy setting of 35%. Ions of unassigned charge states were excluded from fragmentation selection, and the masses of fragmented precursors were dynamically excluded for 25 s. MS/MS transients were acquired at a resolution of 15,000 FWHM using AGC target values and maximum injection of 1e^5^ and 54 ms, respectively.

Data were processed using MaxQuant software (version 1.6.5.0)^66^. Technical replicates (*n* = 3) were merged, while biological replicates were kept separate for statistical analysis. If not mentioned otherwise, standard software settings were kept. The database contained all proteins of the *E. coli* proteome (MG1655, uniprot database) augmented with known lab contaminants. Carbamidomethylation was used as a fixed modification, and methionine oxidation and N-terminal acetylation as variable modifications. Identifications were matched between the runs and were filtered using a target decoy approach at a false discovery rate of 0.01. Biological replicates were compared by LFQ using the MaxQuant LFQ algorithm^67^. To identify Str-induced changes in the proteome the LFQ values were further analyzed in Perseus^68^ with the following parameters. To allow for confident quantifications, a cut-off filter was applied and only proteins that were found in ≥60% of all runs were kept for analysis. Missing values were replaced by random numbers that are drawn from a normal distribution which is modeled on the distribution of the experimental data (default values: width 0.3, downshift 1.8). Significantly altered proteins were identified by ANOVA statistics applying a permutation-based FDR (S0 = 4; FDR 0.05, 250 randomizations). Intensities of significantly regulated proteins were normalized to their z-score and biological replicates were averaged. Co-regulated proteins were identified by hierarchical clustering according to their Euclidian distance. The enrichment of biological processes in the individual clusters was determined by applying Fisher’s exact test. One cluster contained proteins that were significantly upregulated with increasing Str concentrations comprising chaperones and proteases of the heat-shock response. The LFQ-values of these co-regulated chaperones and proteases were scaled to an interval (0–1).

### Quantification of peptides with single amino acid substitutions by DDA

Cells (1 OD_600_) were opened in an SDS loading buffer. Proteins (corresponding to 0.1 OD_600_ cells) were separated by 10% Criterion TGX SDS PAGE (BIORAD). Protein bands of interest were excised from the gel, and the proteins were in-gel proteolyzed with trypsin. Samples were analyzed on an Ultimate 3000 RSLC system coupled to a QExactive HF-X hybrid Quadrupole-Orbitrap mass spectrometer. Tryptic peptides were loaded on a C18 precolumn (2.5 cm, 150 µm ID, Reprosil-Pur 120 Å, 5 µm). Bound peptides were eluted and separated on a C18 capillary column (31 cm, 75 µm ID, packed with Reprosil-Pur 120 Å, 1.9 µm) at a flow rate of 300 nl/min, with a 76 min linear gradient from 5 to 42% ACN in 0.1% formic acid.

Acquisition was performed using two acquisition schemes to maximize identifications while keeping consistent quantifications. Quantification runs were performed in positive ion mode using a top ten DDA method with two micro scans per MS spectrum. MS survey spectra were acquired at a resolution setting of 120.000 FWHM in the range of 350–1600 *m*/*z*, using AGC target values and maximum injection of 3e^6^ and 100 ms, respectively. Precursors with charge states *z* = 2–7 above threshold intensity of 3.0 e^3^ were selected at an isolation width of 1 *m*/*z* for fragmentation by higher energy collision dissociation (HCD) with a normalized collision setting of 35%. Ions of unassigned charge states were excluded from fragmentation selection, and the masses of fragmented precursors were dynamically excluded for 15 s. MS/MS transients were acquired at a resolution setting of 15.000 FWHM using AGC target values and maximum injection of 1e^5^ and 150 ms, respectively.

To gain additional misreading peptide identifications that are aligned to the quantification runs, two additional acquisition schemes were applied using the same chromatography setting. First, a top 30 DDA method using survey scans at a resolution setting of 60.000 FWHM was applied for the analysis of 8 µM and 16 µM Str-treated cell cultures. Second, we applied DDA with MS gas-phase fractionation (350–600; 650–850; 850–1600 *m*/*z*). These additional acquisition schemes, however, provided not more than 5% additional unique misreading peptide identifications.

Acquired data were processed using MaxQuant software (Version 1.6.5.0)^66^. We constructed two sequence databases to allow for the systematic detection of sequence variants. In a first search, a database containing the UniProtKB *E. coli* MG1655 reference proteome (4.491 entries) augmented with known lab contaminants was used. In the main search, EF-Tu peptide sequences (24 cognate peptides) with all possible amino acid substitutions were added to the database (~6300 substituted peptides). The main search mass tolerance was set to 6 ppm. Carbamidomethylation was used as a fixed modification. Peptide identifications were filtered using a target decoy approach at a false discovery rate of 0.01. Typically, between 700 and 900 substituted peptides were identified and further validated. To achieve consistent quantifications the dataset was further analyzed in Skyline (Version 4.2.0)^69^. MaxQuant identifications were imported and the MS signal of the precursor ions (*z* = 2–5) of correct and missense peptides was extracted at a resolution setting of 120.000. Amino acid substitutions were considered to be identified by the globally highest-scoring identification, then quantified by integration over the same elution window in all quantification runs. For some amino acid substitutions, these consistent integration windows led to infinitely low error frequencies due to the absence of detectable peaks or noise. Thus, small constant values were imputed in these cases. Alternatively, (e.g., upon quantification of error clusters in *ram* cells) the use of a consistent integration window in the corresponding chromatographic runs can lead to an integration over the noise in the MS1 space. In such cases, the measured *E*_*f*_ represents an upper limit of the true error frequency.

If a cognate tryptic peptide did not give rise to reliable quantifications, all corresponding substituted peptides were excluded from the analysis. Also, identifications that could not be assigned to any peak or to peaks that did not reach ion-dot products of ≥0.9 (even under induced conditions) were excluded from the analysis. In addition, atryptic peptides, missed cleaved peptides, and identifications with a mass error of ≥3 ppm (even under induced conditions) were excluded from the analysis. As a rule, non-cognate substitutions were excluded from analysis, and in ambiguous cases, amino acid substitutions were interpreted as results of near-cognate misreading. To further reduce false-positive identifications due to the chemical background (i.e., peptides that can be explained by isobaric oxidations or deamidations), we removed all identifications whose signal intensities were not consistently induced (> twofold) either by Str-treatment or in *ram* cells (both relative to the untreated *wt* cells). This filtering resulted in typically 200–300 identifications of induced miscoding events. Previously, we have shown that this procedure diminishes false-positive identifications and improves the quality of the dataset^10^. In some cases, individual features were assigned to different isobaric amino acid substitutions (e.g., A → S, F → Y). Because such regiomers often co-elute and lead to chimeric spectra, this problem is difficult to resolve by DDA approaches on the MS level. However, a stricter filtering that assigns only the amino acid substitution with the highest scoring identification to the feature had no impact on our conclusions. Because Leu and Ile cannot easily be distinguished in standard proteomics workflows, M → I/L and F → I/L substitutions were ambiguous. Using AQUA peptides we validated that AGA induces preferentially M → I and F → L mismatches, which is consistent with the notion that AGA induces third position codon misreading.

Error frequencies were calculated as the ratio of the integrated intensities of the substituted peptide and its correct parental peptide. In a few cases (e.g., when Arg and Lys were substituted or introduced and thus the tryptic cleavage site was removed) no parental peptide was detected (presumably because it was either not fragmented or excluded due to its small size in the database search). In these cases, the median intensity of the correct peptides was used.

To cluster amino acid substitutions into misreading events induced at low and high Str concentrations (Supplementary Fig. 2a) the integrated areas were exported from Skyline^69^ and normalized in Perseus (version 1.6.5.0)^68^. The intensities of each amino acid substitution in different biological states were normalized to the interval from 0 to 1. Reference profiles were used to identify the 50 most similar, regulated amino acid substitutions.

### Identification of error clusters by DDA-MS

Peptides with error clusters were initially identified using the PEAKS software (version 10.5) applying the SPIDER algorithm^70^. The database contained the UniProtKB *E. coli* MG1655 reference proteome and 314 potential modifications in-built in the software were taken into account. The precursor mass tolerance was set to 10 ppm and the fragment mass tolerance to 0.02 Da. Peptide identifications were filtered using a target decoy approach with decoy fusion at a false discovery rate of typically 2% and a typical mutation ion intensity ≥2%. Typical AGA-induced misreading events were weighted by adjusting the mutation-weight matrix; i.e., the likelihood of R → C, N → K, D → E, D → H, C → R, E → D, Q → H, G → C, H → Q, I → M, I → F, I → V, L → Q, L → F, K → N, M → I, F → I, F → L, F → S, S → T, T → S, Y → N, and Y → H was set to −1.882, the value of V → I substitutions. Cluster candidates identified by PEAKS and error clusters that were considered likely to occur because they include combinations of frequent single errors, and the respective peptides with single substitutions, were included in the MaxQuant database described above. MaxQuant results were further analyzed in Skyline^69^. Missense clusters that were induced by Str were manually validated by inspecting the mass accuracy of the precursor, the ion-dot product of the precursor isotope distribution, and the consistency of the highest-scoring MS/MS spectrum with predicted PROSIT spectra^71^ (see Supplementary Fig. 3a). The existence of error clusters was confirmed by PRM and co-eluting isotope-labeled reference peptides.

In some cases, the same amino acid substitution can occur at different positions in a peptide. This can result in regioisomers that often co-elute (see Supplementary Fig. 3a: spectra with red asterisk). These isobaric peptides also co-fragment resulting in chimeric spectra with lower-scoring peptide-spectral matches, which hampers their identification. When such peptides are quantified, we assume that different regioisomers contribute equally to the MS1 signal (Supplementary Fig. 3b, c, open circles). Notably, in PRM experiments the choice of interference-free pseudo-transitions allows dissecting co-eluting, isobaric peptides with error clusters.

To detect error clusters in protein aggregates, an intensity-based validation (as described above) is not possible, because aggregation-prone misreading events are selectively enriched. Thus, we considered only those error clusters that comprise types of errors that were previously validated by the intensity-based approach (due to the limited validation these error clusters are not listed in the Source data file and are not included in Fig. 2g). We note that for samples with high error loads the enrichment of destabilizing errors can lead to an increased fraction of stochastic error clusters. Therefore the estimation of true frequency and origin of destabilizing error clusters in aggregates is obscured and we, therefore, used the data only to estimate the impact of amino acid substitutions on protein stability.

### Selection and quantification of error clusters by targeted MS

A comprehensive proteome-wide analysis of error clusters is not possible due to dynamic range restrictions of mass spectrometry and the high false-positive rate of the analysis. Error clusters reported here were selected for targeting if the peptides for all individual misreading events in the cluster were detected by DDA and these errors were induced by AGA treatment. Only error clusters with net delta mass ≠ 0 and different from known PTMs and peptide derivatizations were considered. In DDA experiments apparent error clusters that were not induced by Str treatment were excluded from the analysis. For targeted experiments, subsets of error clusters were chosen based on sequence distance and physico-chemical properties of the respective peptides (e.g., hydrophobicity). All error clusters that were selected for targeting and that could be unambiguously identified were included in the analysis. The frequency of error clusters was estimated by LFQ and absolute quantification (see Figure captions and the Source data file) in the following way.

In LFQ, correct peptides, peptides with single substitutions, and peptides with error clusters were targeted by PRM in the same sample. Peptides with single substitutions and correct peptides were identified by the high sequence coverage of their co-eluting ion fragments and their high dot product derived from the comparison to spectra identified by search engine runs. In ambiguous cases, AQUA peptides were used to validate the identity of peptides with single substitutions. For the detection of peptides with error clusters, we spiked-in isotope-labeled reference peptides (estimated ≤10 fmol on column, JPT-L). These peptides, which are not used for quantification, help to select interference-free pseudo transitions and to identify the missense peptides by their close-to-one ratio-dot product. Identical amounts of sample were analyzed and controls of non-treated cells were performed to exclude false-positive identifications. LFQ data were only used to estimate error frequencies if the substitutions did not dramatically alter the physico-chemical properties of the peptides (e.g., D → E, E → D, F → L). If clusters involved amino acid substitutions that led to changes in the charge state distribution (e.g., Y → H) or changes in their tryptic cleavage pattern (e.g., R → C, N → K, K → N), measured intensities were not directly interpreted as abundances (Supplementary Figs. 7b (panel 2) and 8). To include such data in the analysis of the distance dependence of error clusters and to minimize the impact of ionization differences, $E_f^{\mathrm{next}}$ values for various AGA were normalized to the $E_f^{\mathrm{next}}$ of Str.

For the absolute quantification of error clusters, we applied the QRAS workflow^10^ with minor changes. QRAS relies on the chromatographic enrichment of target missense peptides in different, partially orthogonal chromatographic steps. After enrichment, target missense peptides are detected and quantified by SRM or PRM. In detail, chromosome-encoded EF-Tu with a C-terminal His-tag was purified and digested under denaturing conditions in 2 M urea. The volume of the tryptic digest was determined and 3–4 tryptic EF-Tu peptides were used to quantify the absolute amount of proteolyzed EF-Tu using AQUA peptides^10^. Sub-stoichiometric amounts of AQUA peptides corresponding to missense peptides with one, two, three, or four substitutions were spiked into the tryptic digest, typically at a ratio of 1:1000–10,000. Subsequently, peptides in the digest were separated in three chromatographic steps: (1). By size-exclusion chromatography (SEC) on a Superdex Peptide 10/300 GL column in an isocratic HPLC run (20% ACN in 0.1% formic acid, (2). By reversed-phase chromatography at neutral pH using a LiChrospher WP300 RP-18 (5 µm) column in an ACN gradient (2–82% ACN in 10 mM ammonium acetate in 45 min), and (3). By reversed-phase chromatography at acidic pH using a LiChrospher WP300 RP-18 (5 µm) column in an ACN gradient (0–65% ACN in 0.1% trifluoroacetic acid in 65 min). In each chromatographic dimension, fractions were screened by SRM to identify target missense peptides. The respective fractions were pooled, concentrated, and applied to the next chromatographic step. After the QRAS workflow, targeted missense peptides are typical ~1000-fold enriched and often among the most abundant peptides in the fraction.

Peptides with error clusters were quantified within the dynamic range of the mass spectrometer by SRM and were further validated by PRM (Supplementary Fig. 4c). To avoid false-positive identifications the potential contamination of AQUA peptides with unlabeled peptides was controlled (in all cases ≤0.1%). In few cases were unlabeled peptide was detected in AQUA peptides, this contamination was taken into account by considering only those quantifications where the signal intensity of the sample exceeded the background level by at least threefold. To illustrate the dependence of error clusters on AGA treatment and to further exclude quantification errors due to light peptide contaminations, the missense peptides derived from treated and untreated cells were enriched by QRAS (Figs. 2b, e and Supplementary Fig. 4b). To allow for a quantitative comparison the loaded sample was normalized by the intensity of the enriched AQUA peptides.

In general, in the analysis of error clusters, the spillover between different chromatographic runs was controlled and was not detectable within the dynamic range of the mass spectrometer.

### Selected reaction monitoring (SRM)

Samples were analyzed on an Ultimate 3000 RSLC system coupled to a TSQ Quantiva triple quadrupole mass spectrometer (Thermo Fisher Scientific). Peptides were separated using an in-house packed column (14 cm, 50 µm ID, packed with Reprosil-Pur C18-AQ 120 Å, 3 µm) at 50 °C. Peptides were eluted in a 45 min linear gradient from 5–50% ACN with 0.1% formic acid at 0.3 µl/min flow rate. Q1 and Q3 were set to unit resolution (0.7 FWHM). A spray voltage of 2100 V was used with a heated ion transfer tube setting of 325 °C. The Chromfilter setting was kept at 3. Scheduled transitions were recorded in a 5 min window and a cycle time of 1 s was applied. Skyline software (Version 3.5) was used for the SRM method setup and data analysis^72,73^. The predominant charge state of each peptide was targeted and 3–5 transitions per peptide were monitored. To ensure high confidence identification, only peptides with ratio dot products ≥0.95 were considered. The light/heavy ratio of each peptide was used for the absolute quantification of correct and missense peptides, which were used to calculate the error frequencies.

### Parallel reaction monitoring (PRM)

Samples were analyzed on an Ultimate 3000 RSLC system coupled to QExactive HF-X hybrid Quadrupole-Orbitrap, QExactive HF hybrid Quadrupole-Orbitrap, or QExactive Plus Hybrid Quadrupole-Orbitrap mass spectrometers (Thermo Fisher Scientific). Acquisition parameters were tuned for the individual experiments and the performance of the mass spectrometer, and can be found in the Source data file. For LFQ, correct peptide ions and peptide ions with amino acid substitutions were targeted in their highest populated charge state. Sets of interference-free fragment chromatograms were extracted using the Skyline software^72,73^ and abundance differences were estimated based on the sum of the individual integrated fragment intensities.

### Quantification and statistical analysis

Error frequencies were estimated as the ratio of missense peptide and correct parental peptide. The expected stochastic error frequency of error clusters was estimated as the product of the error frequencies of the individual misreading events. The amplification over the stochastic level was calculated as the ratio of cluster error frequency and expected stochastic error frequency. The probability of the next misreading event ($E_f^{\mathrm{next}}$) was determined in three different ways (see Supplementary Fig. 7B). (1). $E_f^{\mathrm{next}}$ was derived from time courses and AGA titrations as the slope when the frequency of error clusters was analyzed as a function of the first misreading event (Figs. 4b, d, 5a, and Supplementary Fig. 7a). In addition, $E_f^{\mathrm{next}}$ was estimated from point measurements at a fixed time and AGA concentration. Here, $E_f^{\mathrm{next}}$ was estimated as the ratio of the abundances of the error cluster and the first error. (2). For data generated by absolute quantification and QRAS the ratio of the error frequencies of the error cluster and the first error were used (Figs. 4a, c and Supplementary Fig. 7b, panel 3). (3). Alternatively, peptides with error clusters and their first error were quantified by LFQ and $E_f^{\mathrm{next}}$ was calculated as the ratio of the integrated intensities of peptides with error cluster to peptides with the single first error (Fig. 5b middle and right panels, 5c and Supplementary Figs. 3b, 7b panels 2 and 4, and Supplementary Fig. 8). Notably, the pass criteria for the detection of error clusters, for the analysis of error amplification, and for the analysis of $E_f^{\mathrm{next}}$ differ somewhat (see Supplementary Fig. 3a–c): For the analysis of error amplification correct peptides, peptides with error clusters and with the first and the second error alone have to be quantified. None of the errors should introduce significant changes in physico-chemical properties of peptides, such as charge state, cleavage pattern, or ionizability. For the analysis of $E_f^{\mathrm{next}}$ the pass, criteria are slightly relaxed and only the second error should not introduce significant changes in physico-chemical properties.

Experiments were repeated as indicated in figure legends. Technical replicates are derived from repeats in the analysis of the same sample. Biological replicates derived from analysis of separate, biologically distinct samples produced independently of each other starting from culture inoculation from different clones, and followed by individual cell growth, drug treatment, sample processing, and data acquisition. Statistical analyses were performed with Perseus, Excel, and GraphPadPrism as described in ‘Methods’ and the figure legends. Reported *p* values are based on unpaired *t* tests (two-tailored (Supplementary Fig. 1), and one-tailored (Fig. 1d, f)). When representative results are shown, the experiment has been repeated three times with similar results.

### Reporting summary

Further information on research design is available in the Nature Research Reporting Summary linked to this article.

## Supplementary information

Supplementary Information

Reporting Summary

## Data Availability

Data supporting the findings of this manuscript are available from the corresponding authors upon reasonable request. A reporting summary for this article is available as a Supplementary Information file. Data that support the findings of this study have been deposited to the ProteomeXchange Consortium via the PRIDE and PanoramaPublic repositories. All proteomics data were uploaded to PRIDE repository (in general: PXD019188, data related to Supplementary Fig. 3: PXD022098). Representative DDA-based analysis of amino acid substitutions and targeted MS data that supports all key conclusions was deposited to PanoramaPublic (PXD019328) (https://panoramaweb.org/Error-cluster.url). Spectral evidence for error clusters is presented in the Supplementary Figures. A detailed report on the data structure and availability is given in the Source Data file. Source data are provided with this paper.

## Source data

Source Data
