# Supplementary material for: Translation error clusters induced by aminoglycoside antibiotics
Source: Nat Commun. 2021 Mar 23;12:1830. doi: 10.1038/s41467-021-21942-6 (PMC7987974; doi:10.1038/s41467-021-21942-6)
Supplement: Supplementary file 1 — Supplementary Information [file 41467_2021_21942_MOESM1_ESM.pdf]

Supplementary Information for

Translation Error Clusters Induced by Aminoglycoside Antibiotics

Ingo Wohlgemuth[1], Raffaella Garofalo[1], Ekaterina Samatova[1], Aybeg Nafiz Günenç[1], Christof Lenz[2,3], Henning Urlaub[2,3], and Marina V. Rodnina[1]

[1]Department of Physical Biochemistry, Max Planck Institute for Biophysical Chemistry, Am Fassberg 11, 37077 Goettingen, Germany

[2]Bioanalytical Mass Spectrometry Group, Max Planck Institute for Biophysical Chemistry, Am Fassberg 11, 37077 Goettingen, Germany

[3]Institute of Clinical Chemistry, Bioanalytics, University Medical Center Goettingen, Robert-Koch-Straße 40, 37075 Goettingen, Germany

Correspondence and requests for materials should be addressed to I.W. (email: ingo.wohlgemuth@mpibpc.mpg.de), or to H.U. (email: henning.urlaub@mpibpc.mpg.de), or to M.V.R. (email: rodnina@mpibpc.mpg.de)

Supplementary Table 1

REAGENT or RESOURCE	SOURCE	IDENTIFIER
**Bacterial and Virus Strains**		
Escherichia coli (MG1655)	German Collection of Microorganisms and Cell Cultures	DSM 18039
Escherichia coli RAM cells: UD 131 (Xac rpsD12)	Hani Zaher (Washington University, MO, USA)	
Escherichia coli Corresponding *wt*: Xac (araΔ[lacproAB]ryrA rpoB argEamber)	Hani Zaher (Washington University, MO, USA)	
Escherichia coli with chromosome-encoded C-terminally His-tagged EF-Tu (based on W3110 (K12))	Garofalo et al., 2019	
**Chemicals, Peptides, and Recombinant Proteins**		
RapiGest	Waters	186002123
DTT (Cleland´s Reagent ULTROL Grade)	Millipore	233153 LOT#2758194
Iodoacetamide (BioUltra)	SIGMA	I1149, LOT#SLCC6164
Trypsin (Sequencing Grade modified)	Promega	V5111
AQUA peptides (Ultimate and QuantPro Grade)	ThermoFisher	
Isotope labeled reference peptides for the identification of missense peptides: SpikeTidesL	JPT	
Antibiotics: Amikacin	SIGMA	A2324 LOT#SLBT0718
Antibiotics: Apramycin	LKT Laboratories, Inc	A6264 LOT#23925408
Antibiotics: Apramycin	ChemCruz	sc 210832 LOT#E1217
Antibiotics: Apramycin	Alfa Aesar	J63874 LOT#Z13A011
Antibiotics: Apramycin	SIGMA	LOT# BCBP2820V
Antibiotics: Apramycin	SIGMA	A2024 LOT# 125M4752V
Antibiotics: Dihydrostreptomycin	SIGMA	D7253 LOT#049M4879V
Antibiotics: G418	SIGMA	G8168 LOT#128M4752V
Antibiotics: Gentamycin	SIGMA	G1264 LOT#SLBG7734V
Antibiotics: Hygromycin B	SIGMA	H3274 LOT#SLBH4425V
Antibiotics: Kanamycin A	SIGMA	K4000 LOT#SLBB0945V
Antibiotics: Kanamycin B	SIGMA	B5264 LOT#025M4785V
Antibiotics: Neamine	ChemCruz	Sc 338357 LOT #H0516
Antibiotics: Neomycin	SIGMA	N6386 LOT#SLBD4448V
Antibiotics: Paromomycin	SIGMA	P5057 LOT#118M4116V
Antibiotics: Sisomycin	SIGMA	S7796 LOT#SLBV1849
Antibiotics: Spectinomycin	SIGMA	S9007 LOT#122K0561
Antibiotics: Streptomycin	SIGMA	S9137 LOT#SLBR8944V
Antibiotics: Tobramycin	SIGMA	T1783 LOT#SLBL3840V
**Critical Commercial Assays**		
Protino IDA	Macherey-Nagel 186002123	
**Deposited Data** (see Source data file)		
All Proteomics data	PRIDE	
All DDA runs for the analysis of missense peptides	PRIDE	
Manually validated aminoglycoside titrations (DDA)	Panorama	
Example PRM data for the $\boldsymbol{E_f^{next}}$ determination by LFQ and AGA titrations and time courses	Panorama	
Example PRM data for the $\boldsymbol{E_f^{next}}$ determination by LFQ and point measurements	Panorama	
Example PRM data for complex error cluster and clusters in different proteins	Panorama	
**Software and Algorithms**		
MaxQuant (version 1.5.5.1; 1.6.0.1,1.6.5.0)	Jürgen Cox, MPI for Biochemistry, Munich	
PEAKS Studio (version 10.5)	Bioinformatics Solutions Inc.	
Perseus (version 1.6.5.0)	Jürgen Cox, MPI for Biochemistry, Munich	
Skyline (daily: mainly version 4.2.0.19009 and 20.1.1.158 and 3.5)	Mike MacCoss, University of Washington	
GraphPad Prism (version 8.3)	GraphPad Software Inc.	
CorelDRAW (version 18.1.0.661)	Corel Corporation	

Supplementary Figures

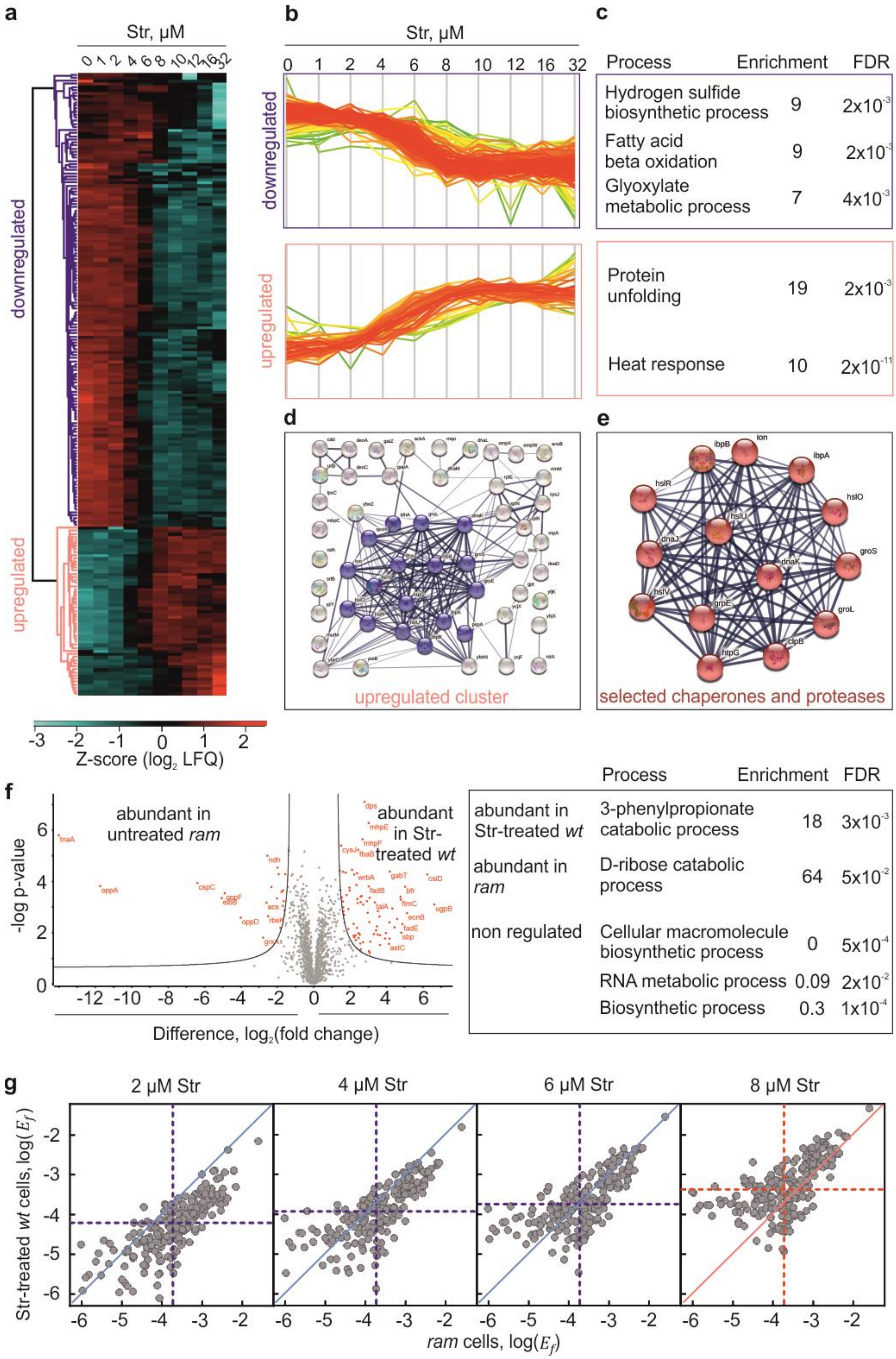

Supplementary Fig. 1: Changes in the cellular proteome upon Str-treatment. Related to Fig. 1

a Hierarchical clustering of upregulated (red) and downregulated (blue) proteins after treatment of *E. coli* Xac strain (*wt*) with increasing Str concentrations. Protein abundancies in cell lysate were compared by LFQ using the MaxQuant LFQ algorithm[1]. Significantly regulated proteins were identified by a multiple sample ANOVA test and compared by their z-scores. Biological replicates (*n*=3) were averaged and proteins hierarchically clustered by their Euclidean distance.

b Profile view of up- and downregulated proteins.

c Pathway enrichment analysis of individual clusters. False discovery rate (FDR) was corrected by the Benjamini-Hochberg procedure.

d Proteins significantly upregulated upon Str treatment (as in a). Members of the heat shock regulon are shown in blue.

e Subset of upregulated proteins: chaperones and proteases of the heat shock response pathway that are chosen to correlate with the error level: ClpB, Lon, HtpG, DnaJ, GrpE, DnaK, IbpB, IbpA, HslU, HslV, HslO, HslR, GroEL, and GroES.

f Left panel: Proteome comparison between untreated *ram* and Str-treated *wt* cells. Samples at a similar stress level were chosen for comparison (4 µM Str). Right panel: Pathway enrichment analysis of individual clusters. FDR was corrected by the Benjamini-Hochberg procedure. Enrichment values <1 indicate that proteins of these processes are significantly underrepresented among the regulated proteins. Proteins involved in biosynthetic processes and macromolecule synthesis have similar level at comparable stress level, consistent with the notion that translation rate and growth rate of *ram* and *wt* cells are similar.

g Error frequencies in Str-treated *wt* and untreated *ram* cells. Single amino acid substitutions were quantified by mass spectrometry in EF-Tu using cell lysates described in (A). Gray points represent individual missense peptides. For each condition, median error frequencies are indicated by dashed lines. Note that due to the low error frequency of most amino acid substitutions and the dynamic range restrictions of the mass spectrometer, the median error frequencies do not reflect the global median error frequency of near-cognate misreading, but are solely used for relative comparison of miscoding in *wt* Str-treated vs. *ram* cells.

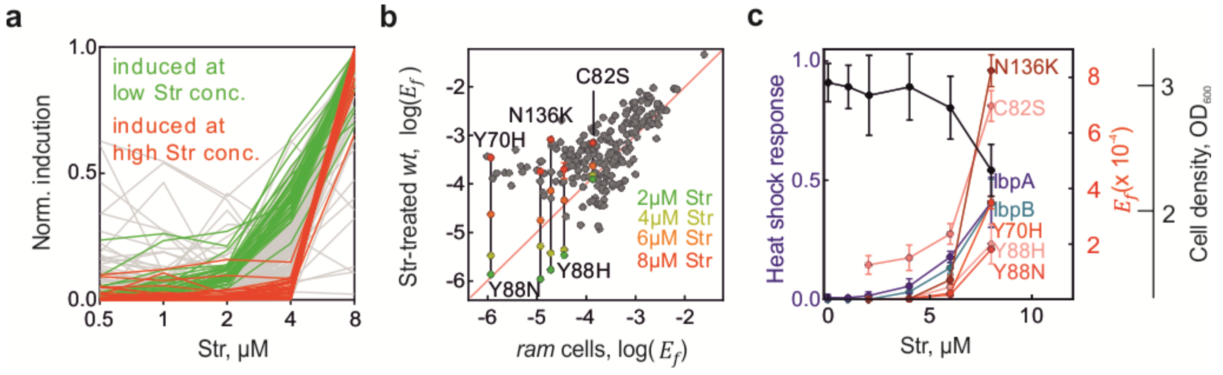

Supplementary Fig. 2: Analysis of Str-induced translation errors (single amino acid substitutions). Related to Fig. 2

a Translation errors induced at low (green) and high (red) Str concentrations. Cells (*E. coli* strain W3110) were treated with increasing Str concentrations for 2 h. EF-Tu was purified using the chromosome-encoded C-terminal tag under denaturating conditions in 8 M urea. For better comparison, peptide intensities were normalized to the interval (0 to 1). Technical replicates were averaged. The 50 errors induced at lowest and highest Str concentrations were identified with Perseus[2] and matched with the data on protein aggregation (Fig. 1d). Those errors that do not belong to one of the two clusters are shown in gray.

b E_f of selected errors that are induced at high Str concentrations, which are associated with an enhanced aggregation propensity mapped on Supplementary Fig. 1g (8 µM Str).

c Visualization of the concerted growth inhibition, induction of small chaperones and appearance of aggregation-prone, late-responding amino acid substitutions. Data replotted from Fig. 1a and Supplementary Fig. 1g; Normalization as in Fig. 1a.

a

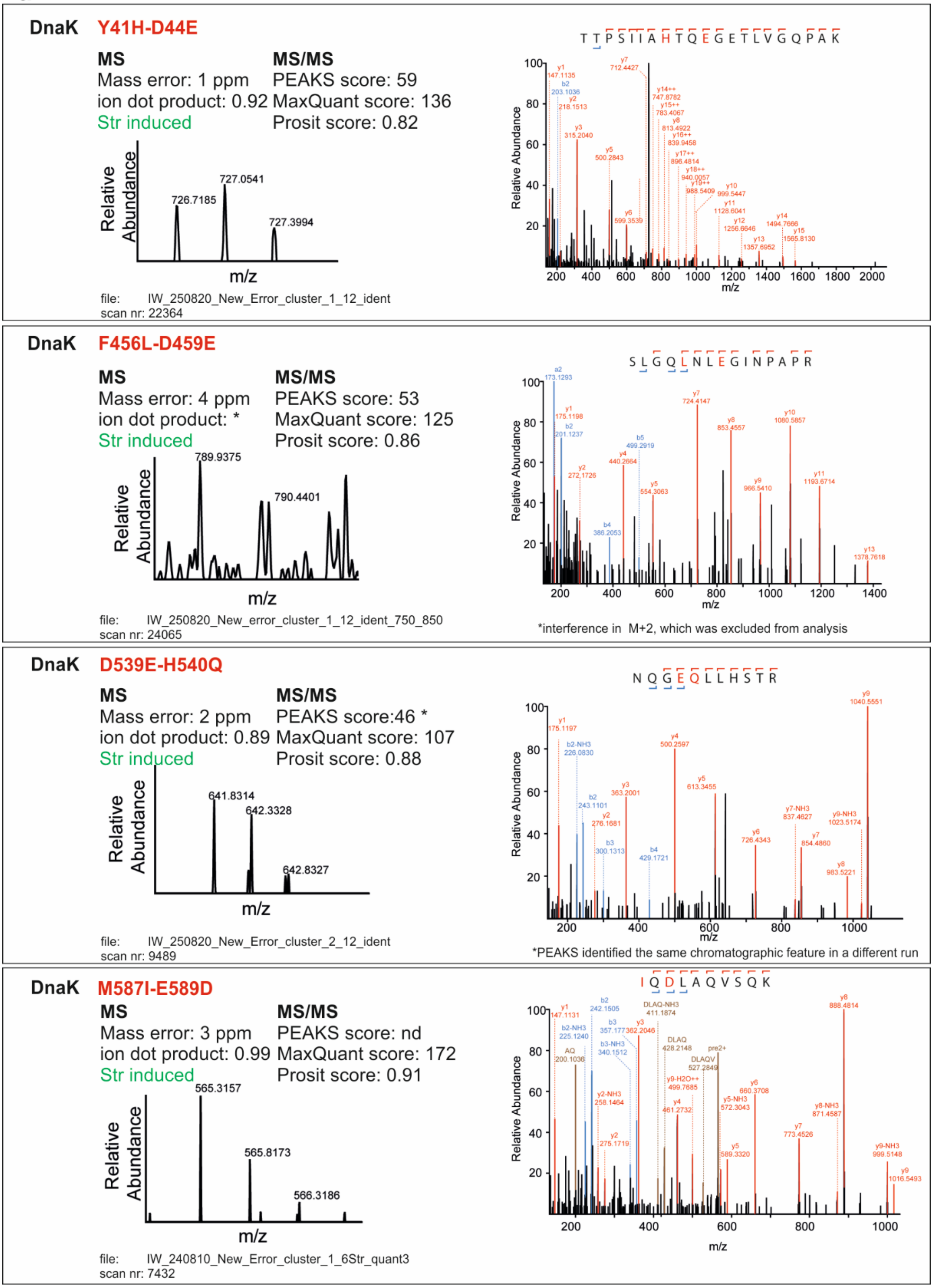
DnaK Y41H-D44E
MS
Mass error: 1 ppm
ion dot product: 0.92
Str induced
MS/MS
PEAKS score: 59
MaxQuant score: 136
Prosit score: 0.82
Relative Abundance
726.7185
727.0541
727.3994
m/z
file: IW_250820_New_Error_cluster_1_12_ident
scan nr: 22364
T T P S I I A H T Q E G E T L V G Q P A K
Relative Abundance
m/z
DnaK F456L-D459E
MS
Mass error: 4 ppm
ion dot product: *
Str induced
MS/MS
PEAKS score: 53
MaxQuant score: 125
Prosit score: 0.86
789.9375
790.4401
file: IW_250820_New_error_cluster_1_12_ident_750_850
scan nr: 24065
S L G Q L N L E G I N P A P R
*interference in M+2, which was excluded from analysis
DnaK D539E-H540Q
MS
Mass error: 2 ppm
ion dot product: 0.89
Str induced
MS/MS
PEAKS score:46 *
MaxQuant score: 107
Prosit score: 0.88
641.8314
642.3328
642.8327
file: IW_250820_New_Error_cluster_2_12_ident
scan nr: 9489
N Q G E Q L L H S T R
*PEAKS identified the same chromatographic feature in a different run
DnaK M587I-E589D
MS
Mass error: 3 ppm
ion dot product: 0.99
Str induced
MS/MS
PEAKS score: nd
MaxQuant score: 172
Prosit score: 0.91
565.3157
565.8173
566.3186
file: IW_240810_New_Error_cluster_1_6Str_quant3
scan nr: 7432
I Q D L A Q V S Q K

EF-Tu **F47L-D51E**

MS
Mass error: 1 ppm
ion dot product: 0.98
Str induced

MS/MS
PEAKS score: nd
MaxQuant score: 123
Prosit score: 0.88

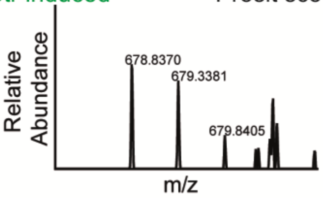

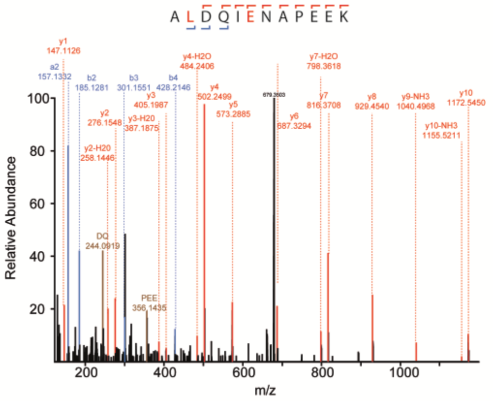

file: I_Wohlgemuth_25092019_8Str_ident
scan nr: 12230

EF-Tu **D48E-D51E**

MS
Mass error: -2 ppm
ion dot product: 0.95
Str induced

MS/MS
PEAKS score: nd
MaxQuant score: 173
Prosit score: 0.89

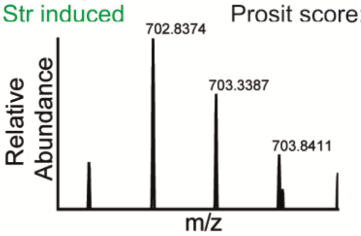

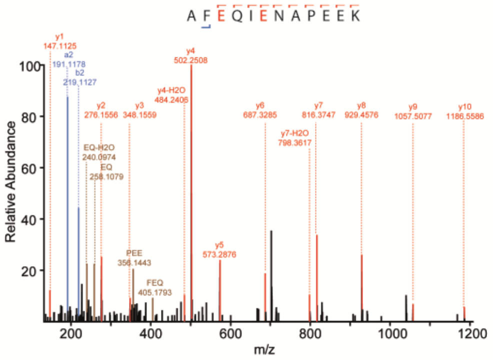

file: I_Wohlgemuth_24092019_8Str_quant3
scan nr: 9315

EF-Tu **D51E-N52K**

MS
Mass error: 1 ppm
ion dot product: 0.96
Str induced

MS/MS
PEAKS score: nd
MaxQuant score: 143
Prosit score: 0.93

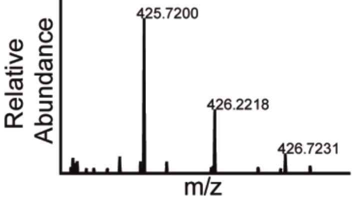

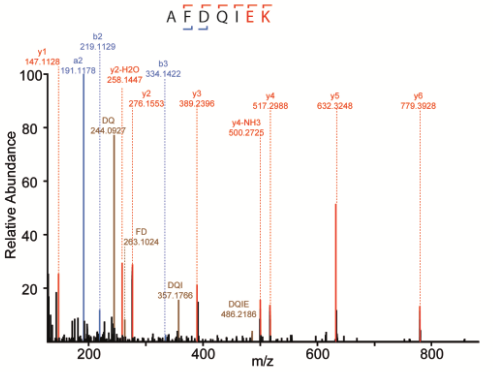

file: I_Wohlgemuth_24092019_8Str_quant1
scan nr: 6746

EF-Tu **H67Q-E69D**

MS
Mass error: 0 ppm
ion dot product: 0.97
Str induced

MS/MS
PEAKS score: 70
MaxQuant score:248
Prosit score: 0.91

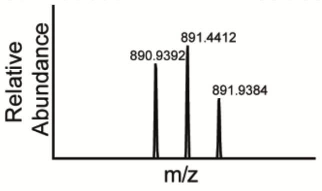

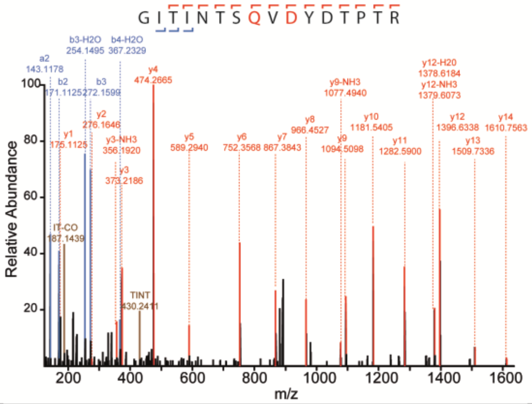

file: I_Wohlgemuth_25092019_16Str_ident
scan nr: 20963

EF-Tu **F163L-D167E**

MS
Mass error: 0 ppm
ion dot product: 0.98
Str induced

MS/MS
PEAKS score: 63
MaxQuant score: 87
Prosit score:0.83

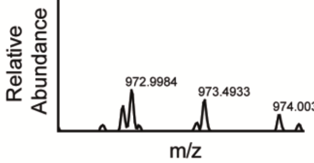

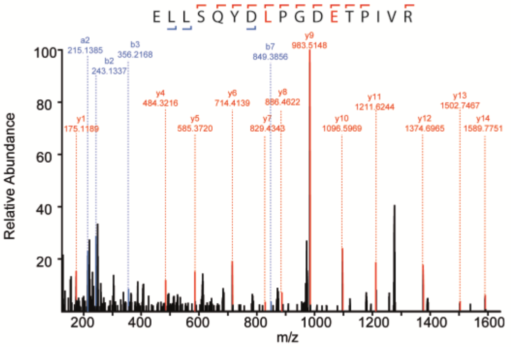

file: I_Wohlgemuth_25092019_8Str_850-1600
scan nr: 20417

EF-Tu **E191D-F195L**

MS
Mass error: 3 ppm
ion dot product: 0.99
Str induced

MS/MS
PEAKS score: 52
MaxQuant score: 148
Prosit score: 0.91

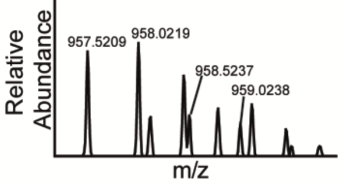

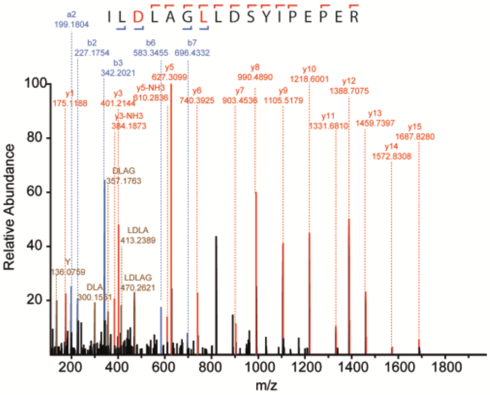

file: I_Wohlgemuth_B209vsB212_rep3_8Str_ident
scan nr: 37372

EF-Tu **E191D-Y199H**

MS
Mass error: 5 ppm
ion dot product: 1
Str induced

MS/MS
PEAKS score: 55
MaxQuant score: 91
Prosit score: 0.91

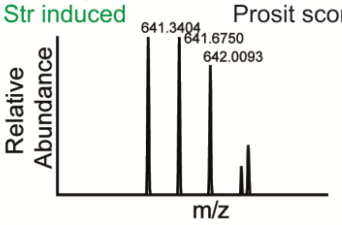

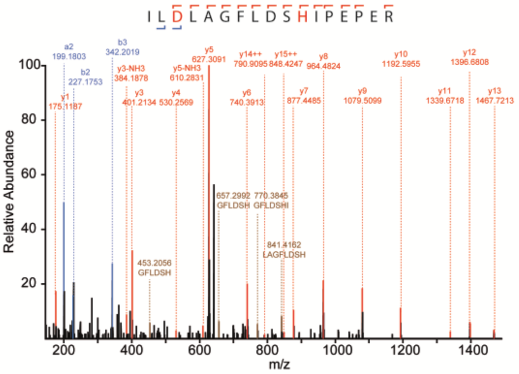

file: I_Wohlgemuth_25092019_8Str_ident
scan nr: 37718

EF-Tu **E191D-E202D**

MS
Mass error: 0 ppm
ion dot product: 0.99
Str induced

MS/MS
PEAKS score: 54
MaxQuant score: 141
Prosit score: 0.88

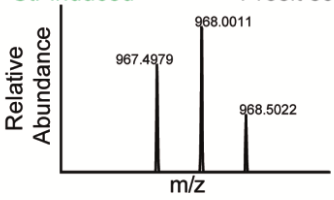

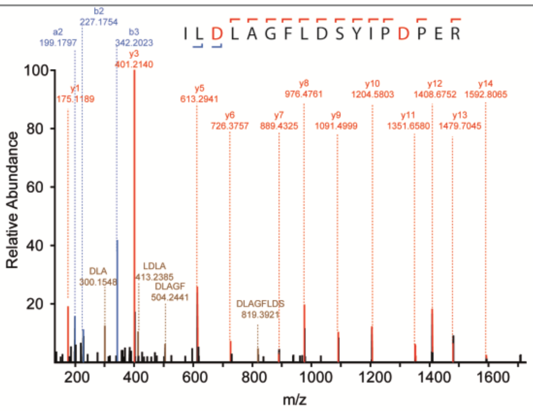

file: I_wohlgemuth_B209vsB212_rep3_8Str_quant1
scan nr: 27407

EF-Tu **F195L-D197E**

MS	MS/MS
Mass error: 2 ppm	PEAKS score: 51
ion dot product: 0.97	MaxQuant score: 90
Str induced	Prosit score: 0.84

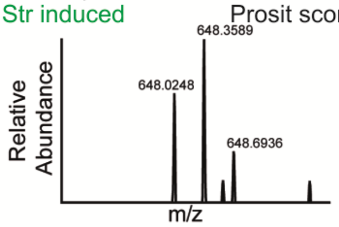

file: 39542
scan nr: I_Wohlgemuth_B209vsB212_rep3_16Str_ident

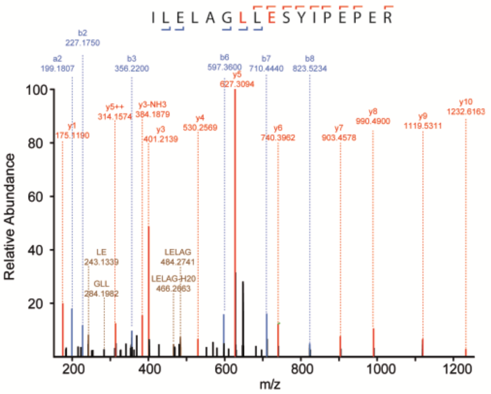

EF-Tu **F195L-Y199H**

MS	MS/MS
Mass error: 2 ppm	PEAKS score: nd
ion dot product: 0.99	MaxQuant score: 54
Str induced	Prosit score: 0.81

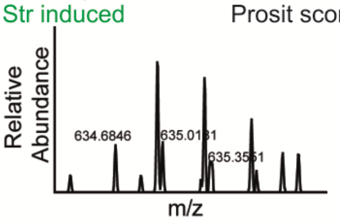

file: IWohlgemuth_260619_16Str_identification
scan nr: 39354

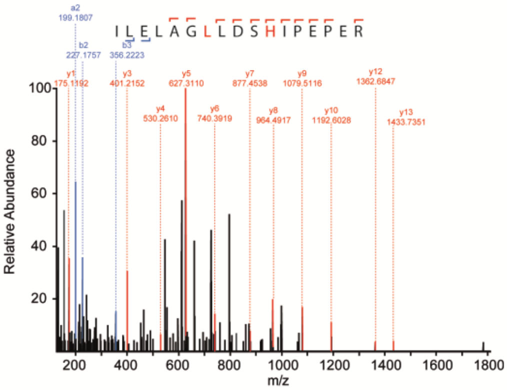

EF-Tu **F195L-E202D**

MS	MS/MS
Mass error: 2 ppm	PEAKS score: nd
ion dot product: 0.86	MaxQuant score: 63
Str induced	Prosit score: 0.76

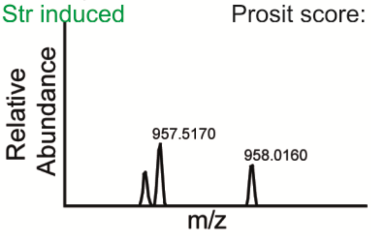

file: IWohlgemuth_260619_8Str_identification
scan nr: 45108

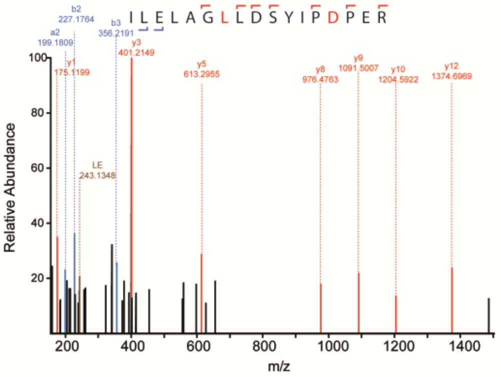

EF-Tu **D197E-Y199H**

MS	MS/MS
Mass error: 0 ppm	PEAKS score: 60
ion dot product: 0.97	MaxQuant score: 125
Str induced	Prosit score: 0.93

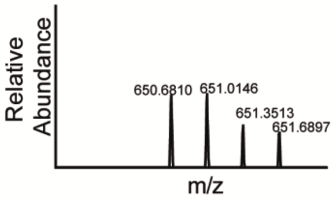

file: I_Wohlgemuth_25092019_8Str_ident
scan nr: 37613

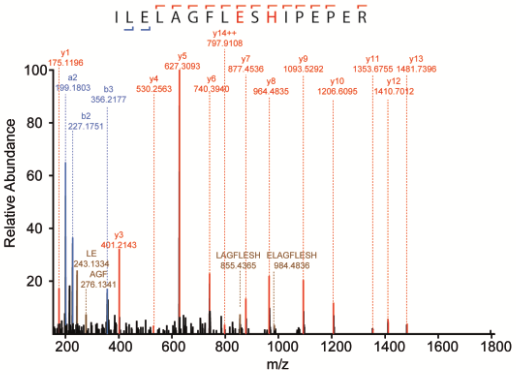

EF-Tu Y199H-E202D

MS	MS/MS
Mass error: 2 ppm	PEAKS score: nd
ion dot product: 0.94	MaxQuant score: 81
Str induced	Prosit score: 0.87

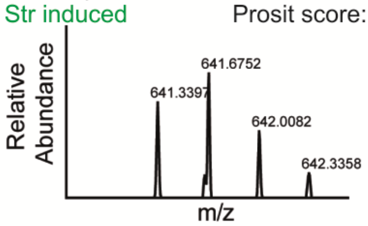

file: I_Wohlgemuth_25092019_16Str_ident
scan nr: 38889

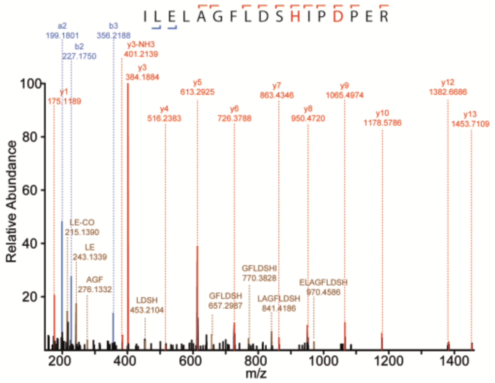

EF-Tu D208E-F211L

MS	MS/MS
Mass error: 1 ppm	PEAKS score: 66
ion dot product: 0.99	MaxQuant score: 96
Str induced	Prosit score: 0.91

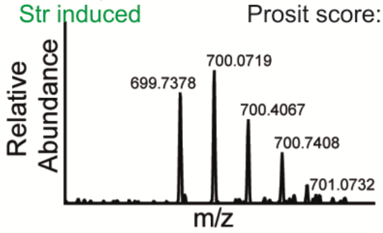

file: I_Wohlgemuth_25092019_8Str_600-850
scan nr: 36367

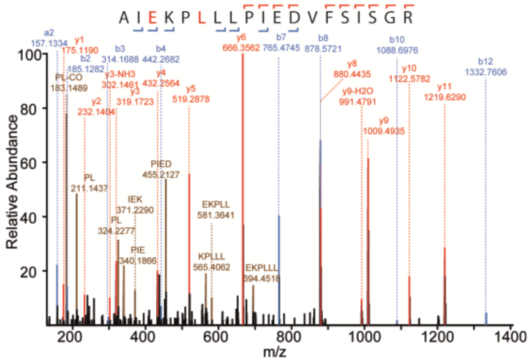

EF-Tu D208E-F219L

MS	MS/MS
Mass error: 3 ppm	PEAKS score: 54
ion dot product: 0.99	MaxQuant score: 115
Str induced	Prosit score: 0.91

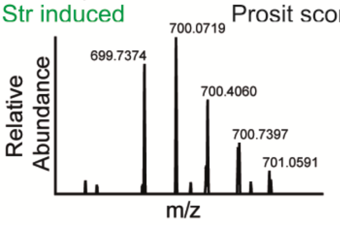

file: I_Wohlgemuth_24092019_8Str_quant1
scan nr: 29193

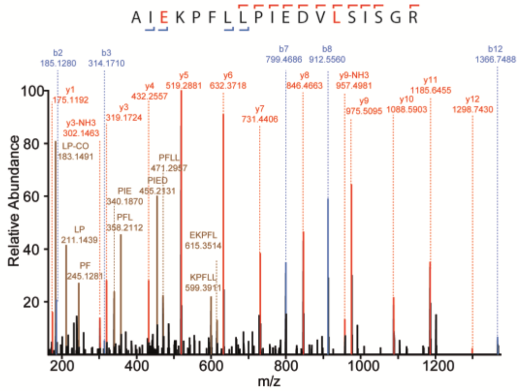

EF-Tu F211L-E216D

MS	MS/MS
Mass error: 3 ppm	PEAKS score: 57
ion dot product: 1	MaxQuant score: 105
Str induced	Prosit score: 0.92

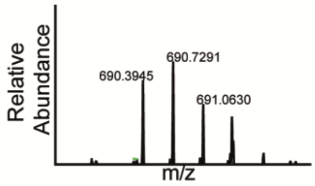

file: I_wohlgemuth_B209vsB212_rep3_8Str_quant2
scan nr: 23554

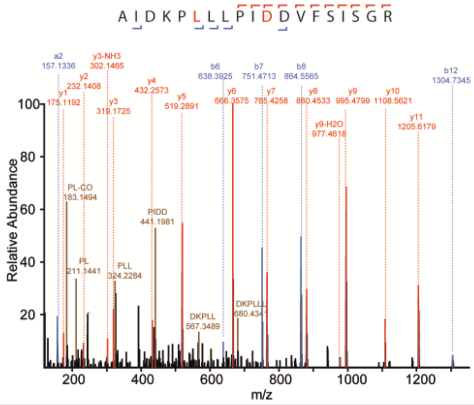

EF-Tu F211L-D217E

MS
Mass error: 2 ppm
ion dot product: 0.94
Str induced

MS/MS
PEAKS score: nd
MaxQuant score: 80
Prosit score: 0.83

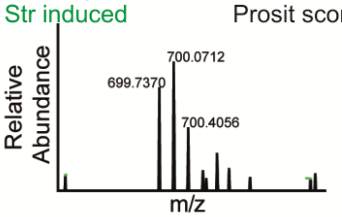

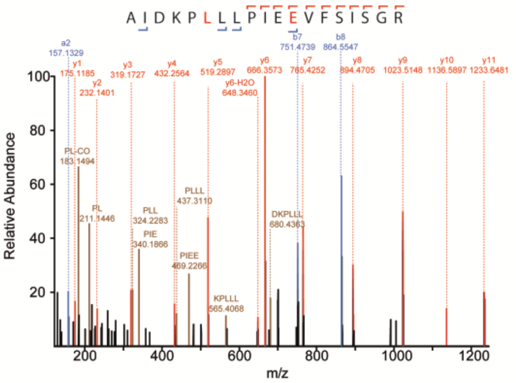

file: I_Wohlgemuth_B209vsB212_rep3_16Str_ident
scan nr: 37760

EF-Tu F211L-F219L

MS
Mass error: 1 ppm
ion dot product: 0.95
Str induced

MS/MS
PEAKS score: 65
MaxQuant score: 119
Prosit score: 0.90

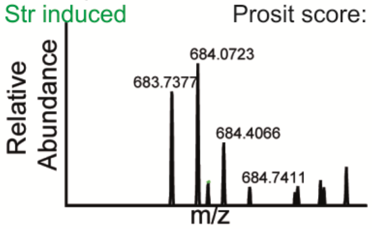

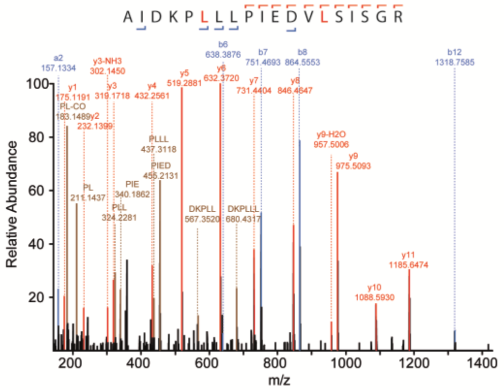

file: I_Wohlgemuth_25092019_8Str_ident
scan nr: 40434

EF-Tu E216D-F219L

MS
Mass error: 1 ppm
ion dot product: 0.97
Str induced

MS/MS
PEAKS score: 59
MaxQuant score: 92
Prosit score: 0.91

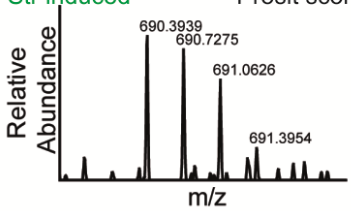

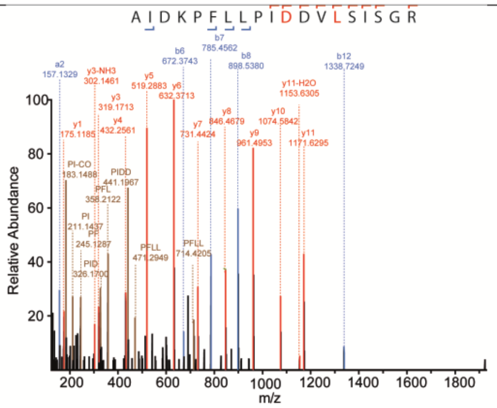

file: I_Wohlgemuth_25092019_8Str_600-850
scan nr: 36366

EF-Tu D217E-F219L

MS
Mass error: 3 ppm
ion dot product: 0.98
Str induced

MS/MS
PEAKS score: 54
MaxQuant score: 96
Prosit score: 0.92

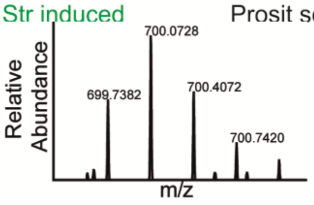

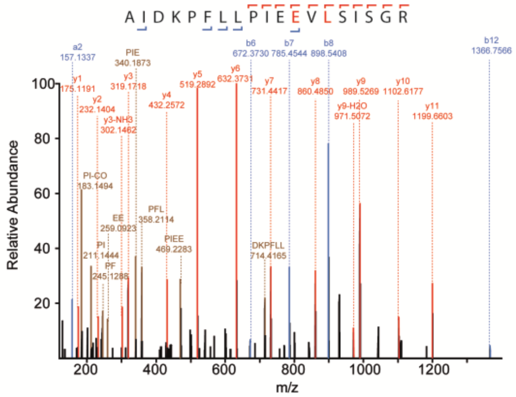

file: I_wohlgemuth_B209vsB212_rep3_8Str_quant2
scan nr: 26567

EF-Tu E241D-E242D

MS	MS/MS
Mass error: 3 ppm	PEAKS score: nd
ion dot product: 0.98	MaxQuant score: 128
Str induced	Prosit score: 0.90

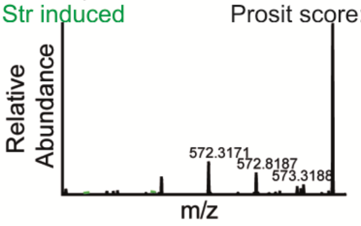

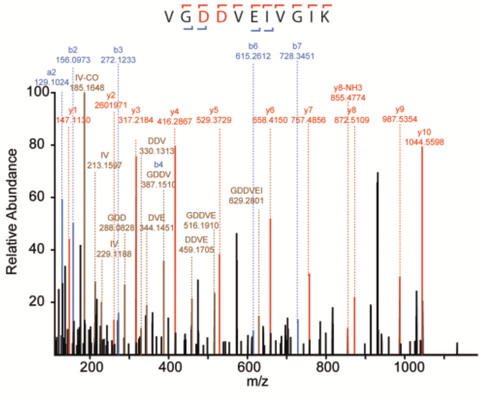

file: I_wohlgemuth_B209vsB212_rep3_8Str_quant1
scan nr: 12188

EF-Tu E241D-E244D

MS	MS/MS
Mass error: -1 ppm	PEAKS score: nd
ion dot product: 0.95	MaxQuant score: 166
Str induced	Prosit score: 0.93

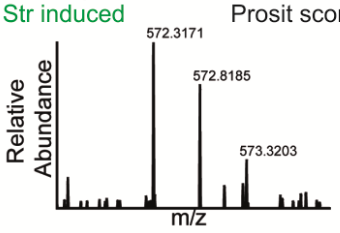

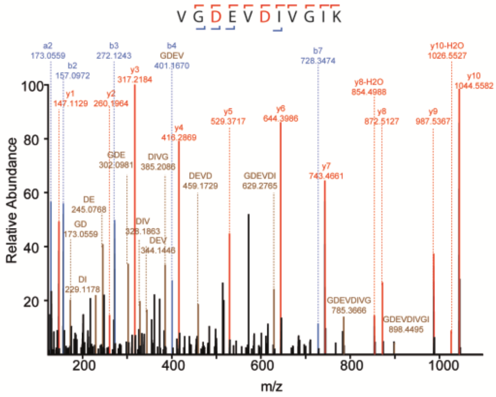

file: I_wohlgemuth_B209vsB212_rep3_8Str_quant2
scan nr: 12535

EF-Tu E242D-E244D

MS	MS/MS
Mass error: 1 ppm	PEAKS score: 41
ion dot product: 0.98	MaxQuant score: 128
Str induced	Prosit score: 0.91

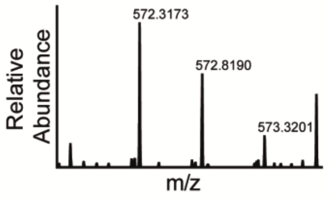

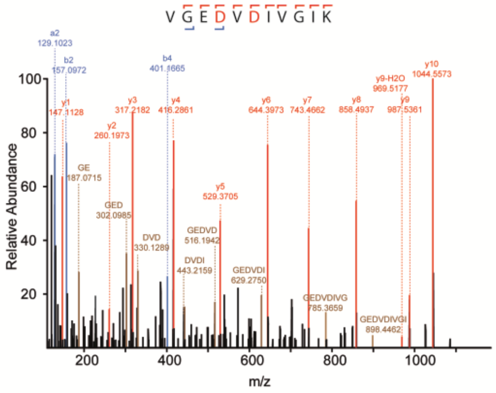

file: I_wohlgemuth_B209vsB212_rep3_8Str_quant1
scan nr: 12473

EF-Tu M262I-F262L

MS	MS/MS
Mass error: 2 ppm	PEAKS score: 26
ion dot product: 0.97	MaxQuant score: 72
Str induced	Prosit score: 0.89

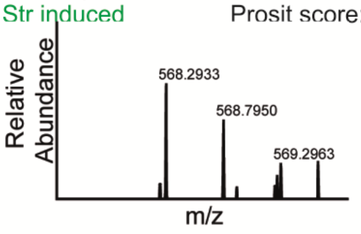

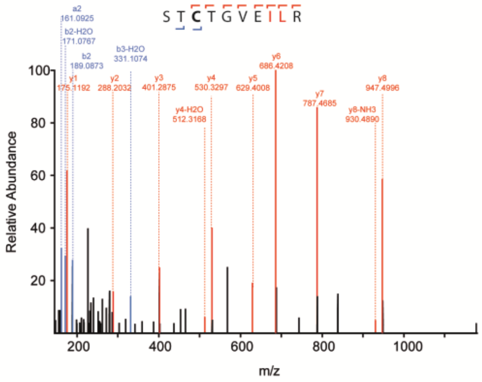

file: IWohlgemuth_250619_8Str_quant3
scan nr: 10533

EF-Tu **M261I-R263C**

MS	MS/MS
Mass error: 2 ppm	PEAKS score: nd
ion dot product: 0.96	MaxQuant score: 93
Str induced	Prosit score: 0.89

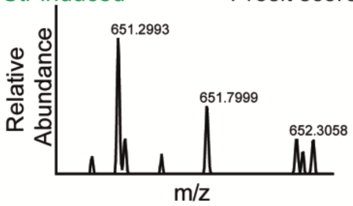

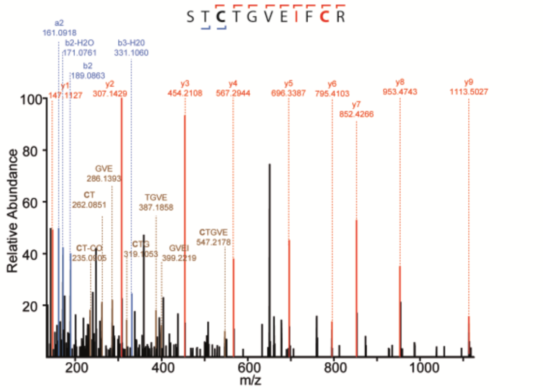

file: I_Wohlgemuth_25092019_8Str_ident
scan nr: 18789

EF-Tu **M359I-H365Q**

MS	MS/MS
Mass error: 3 ppm	PEAKS score: nd
ion dot product: 0.97	MaxQuant score: 158
Str induced	Prosit score: 0.93

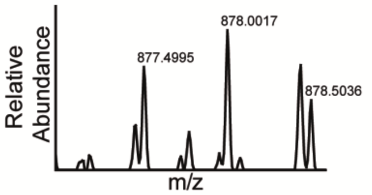

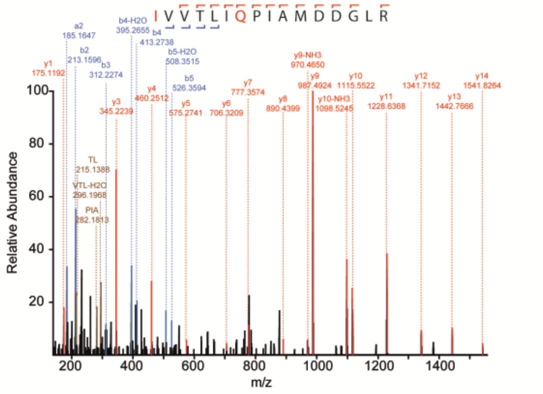

file: I_Wohlgemuth_25092019_8Str_850-1600
scan nr: 26086

EF-Tu **M359I-M369I**

MS	MS/MS
Mass error: 3 ppm	PEAKS score: nd
ion dot product: 0.99	MaxQuant score: 105
Str induced	Prosit score: 0.89

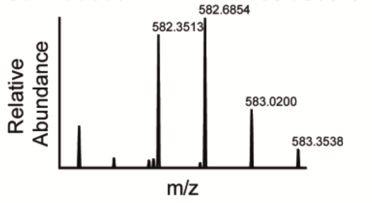

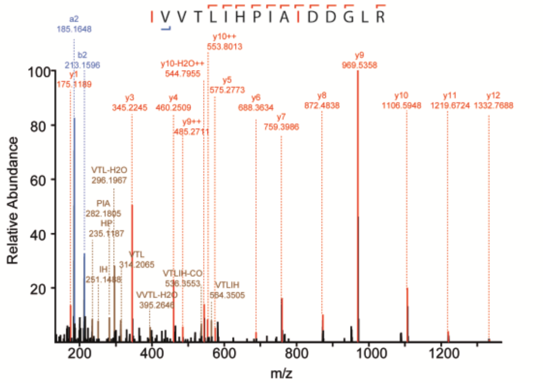

file: I_Wohlgemuth_24092019_8Str_quant1
scan nr: 23257

GAPDH-A **Y138H-C149R**

MS	MS/MS
Mass error: 1 ppm	PEAKS score: 47
ion dot product: 0.92	MaxQuant score: 107
Str induced	Prosit score: 0.82

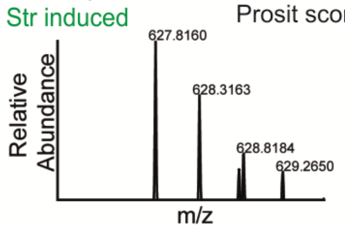

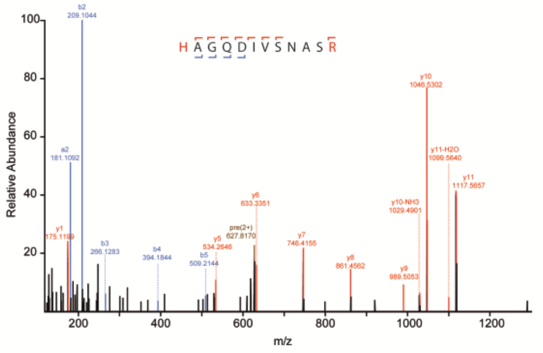

file: IW_240820_New_Error_cluster_2_12_ident
scan nr: 16759

GroL **E128D-E129D**

MS
Mass error: 0 ppm
ion dot product: 0.95
Str induced

MS/MS
PEAKS score: 37
MaxQuant score: 95
Prosit score: 0.9

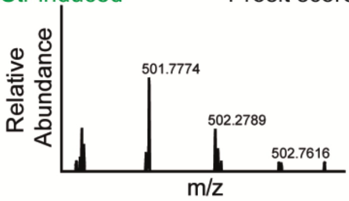

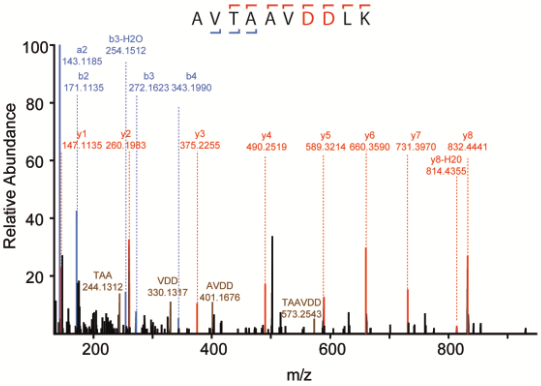

file: IW_270810_New_Error_cluster_4_8Str_quant3
scan nr: 7359

GroL **T148S-E155D**

MS
Mass error: 5 ppm
ion dot product: 0.99
Str induced

MS/MS
PEAKS score: 65
MaxQuant score: 189
Prosit score: 0.85

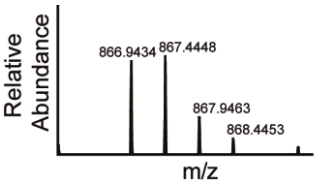

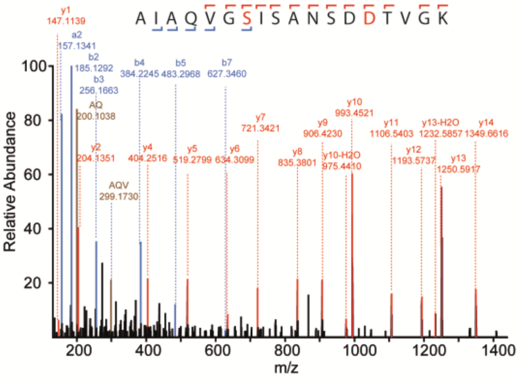

file: IW_270810_New_Error_cluster_4_8Str_quant2
scan nr: 9071

GroL **E396D-H400Q**

MS
Mass error: 3 ppm
ion dot product: 0.98*
Str induced

MS/MS
PEAKS score: 36
MaxQuant score: 151
Prosit score: 0.91

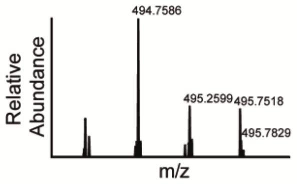

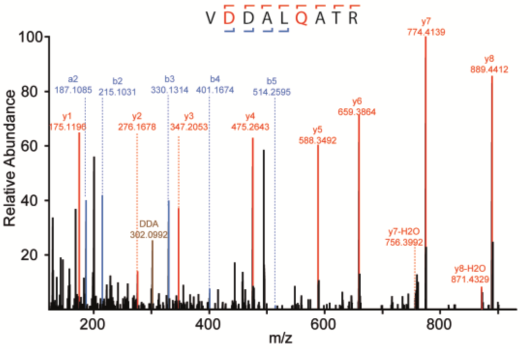

file: IW_270810_New_Error_cluster_4_8Str_quant3
scan nr: 4059

* interference in pre2+2, only first half of the peak analyzed for idotp

OmpA **Y241H-S242R**

MS
Mass error: 2 ppm
ion dot product: 0.99
Str induced

MS/MS
PEAKS score: 47
MaxQuant score: 50
Prosit score: 0.84

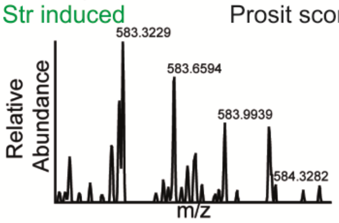

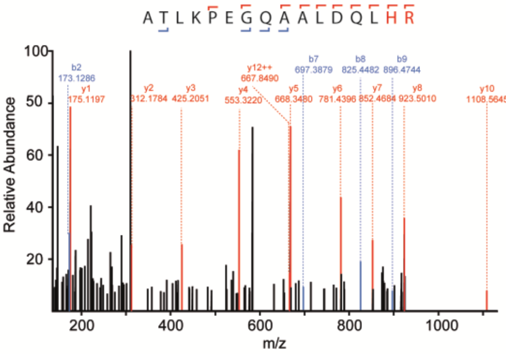

file: IW_270820_New_Error_cluster_3_12_ident_550_650
scan nr: 11337

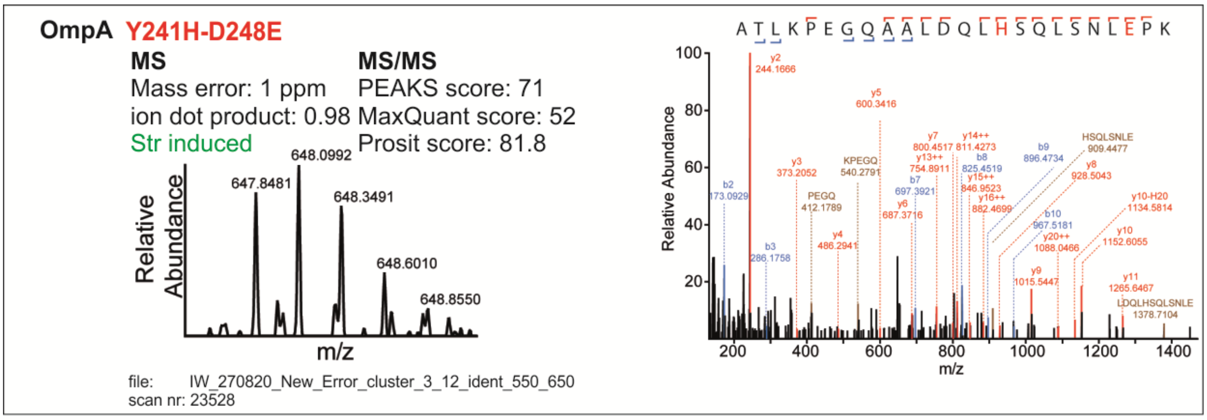
OmpA Y241H-D248E
MS
Mass error: 1 ppm
ion dot product: 0.98
Str induced
MS/MS
PEAKS score: 71
MaxQuant score: 52
Prosit score: 81.8
Relative Abundance
m/z
file: IW_270820_New_Error_cluster_3_12_ident_550_650
scan nr: 23528
A T L K P E G Q A A L D Q L H S Q L S N L E P K
Relative Abundance
m/z

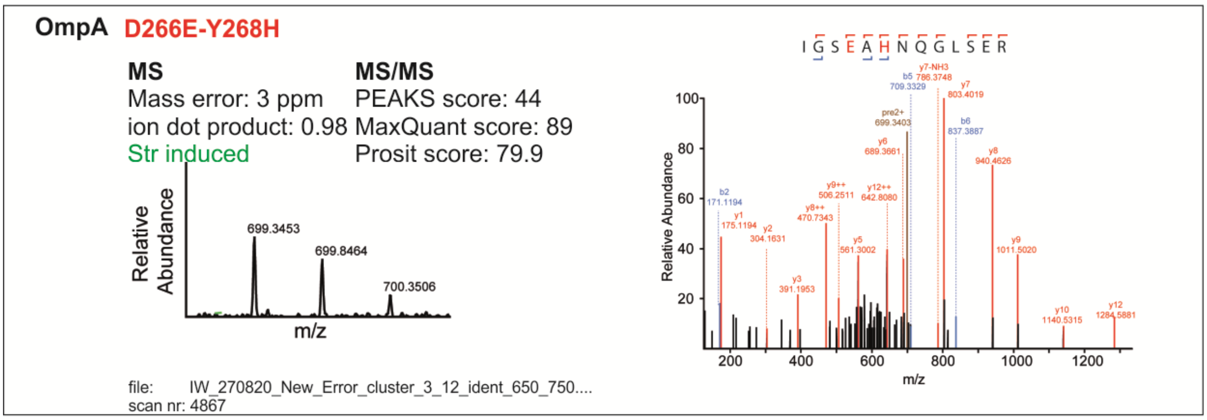
OmpA D266E-Y268H
MS
Mass error: 3 ppm
ion dot product: 0.98
Str induced
MS/MS
PEAKS score: 44
MaxQuant score: 89
Prosit score: 79.9
Relative Abundance
m/z
file: IW_270820_New_Error_cluster_3_12_ident_650_750....
scan nr: 4867
I G S E A H N Q G L S E R
Relative Abundance
m/z

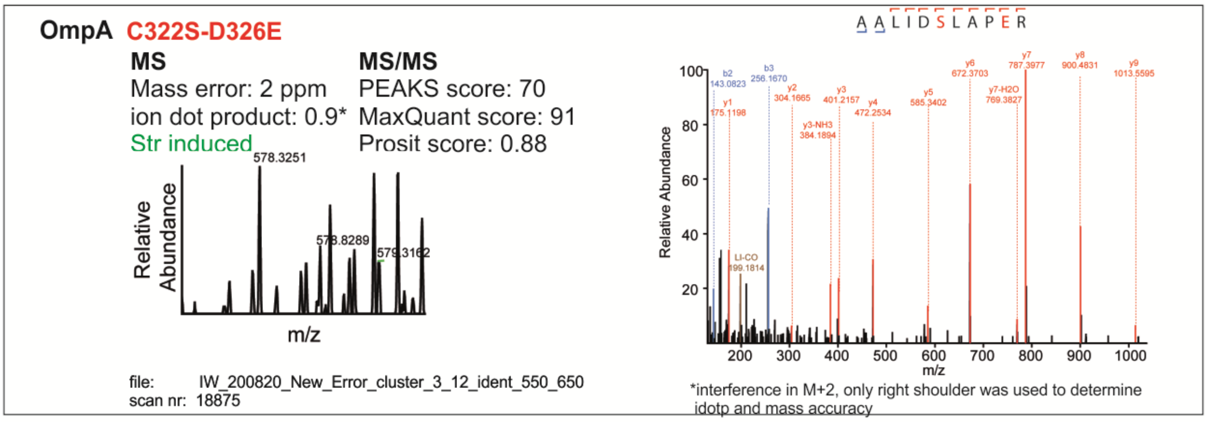
OmpA C322S-D326E
MS
Mass error: 2 ppm
ion dot product: 0.9*
Str induced
MS/MS
PEAKS score: 70
MaxQuant score: 91
Prosit score: 0.88
Relative Abundance
m/z
file: IW_200820_New_Error_cluster_3_12_ident_550_650
scan nr: 18875
A A L I D S L A P E R
Relative Abundance
m/z
*interference in M+2, only right shoulder was used to determine idotp and mass accuracy

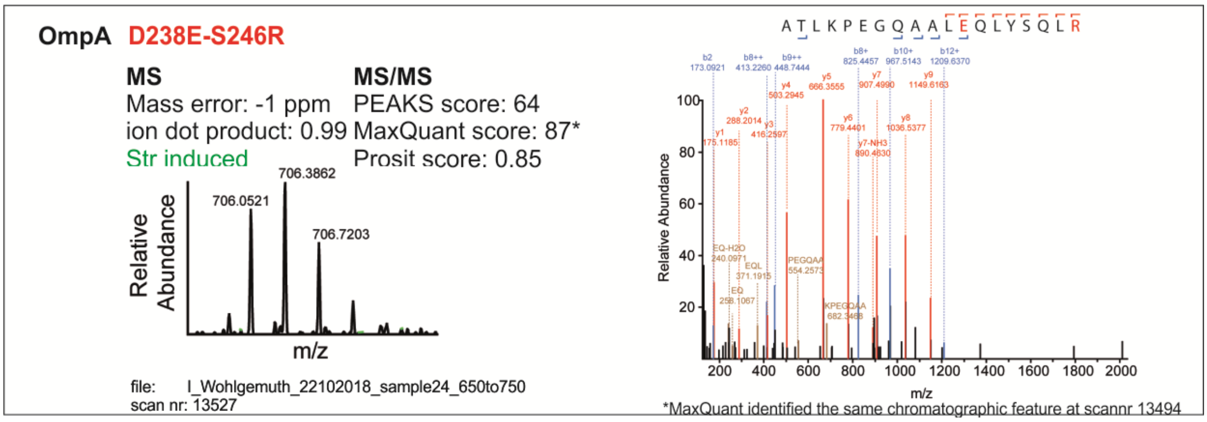
OmpA D238E-S246R
MS
Mass error: -1 ppm
ion dot product: 0.99
Str induced
MS/MS
PEAKS score: 64
MaxQuant score: 87*
Prosit score: 0.85
Relative Abundance
m/z
file: I_Wohlgemuth_22102018_sample24_650to750
scan nr: 13527
A T L K P E G Q A A L E Q L Y S Q L R
Relative Abundance
m/z
*MaxQuant identified the same chromatographic feature at scannr 13494

OmpC Y78H-S84R

MS
Mass error: 6 ppm
ion dot product: 0.96
Str induced

MS/MS
PEAKS score: 67
MaxQuant score: 108
Prosit score: 0.85

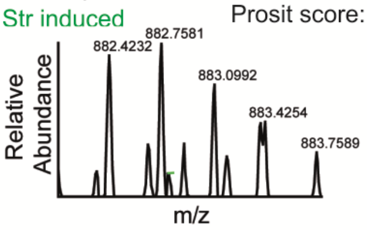

file: IW_250820_New_Error_cluster_2_12_ident_850_1600
scan nr: 22502

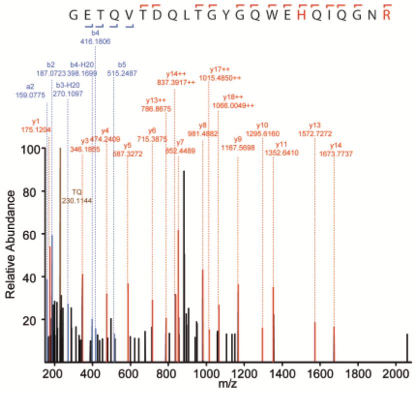

OmpC F102L-D104E
*

MS
Mass error: 2 ppm
ion dot product: 0.99
Str induced

MS/MS
PEAKS score: 53
MaxQuant score: 148
Prosit score: 0.94

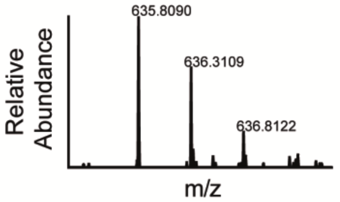

file: IW_240810_New_Error_Cluster_2_8Str_quant_2
scan nr:12515

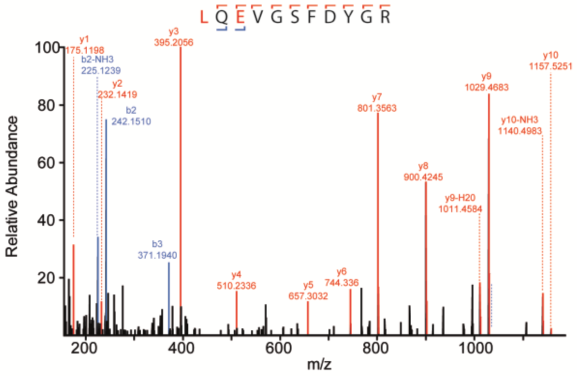

OmpC F102L-D109E
*

MS
Mass error: 1 ppm
ion dot product: 0.99
Str induced

MS/MS
PEAKS score: 53
MaxQuant score: 150
Prosit score: 0.88

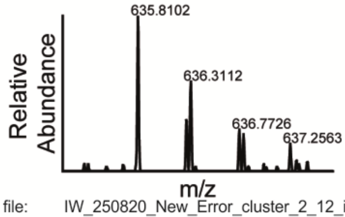

file: IW_250820_New_Error_cluster_2_12_ident_550_650
scan nr: 18558

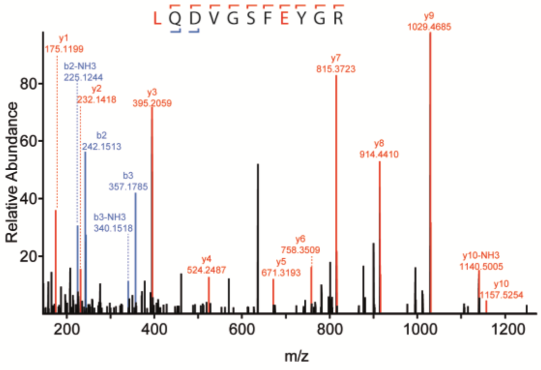

OmpC D104E-D109E

MS
Mass error: 3 ppm
ion dot product: 1
Str induced

MS/MS
PEAKS score: 42
MaxQuant score: 97
Prosit score: 0.89

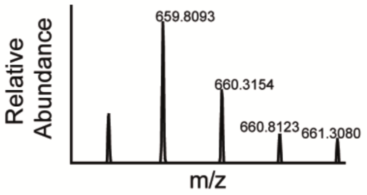

file: IW_250820_New_Error_cluster_2_12_ident
scan nr: 24524

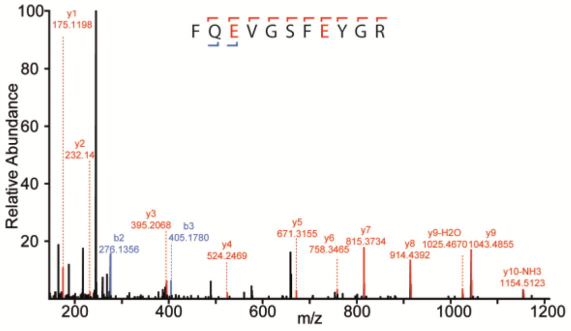

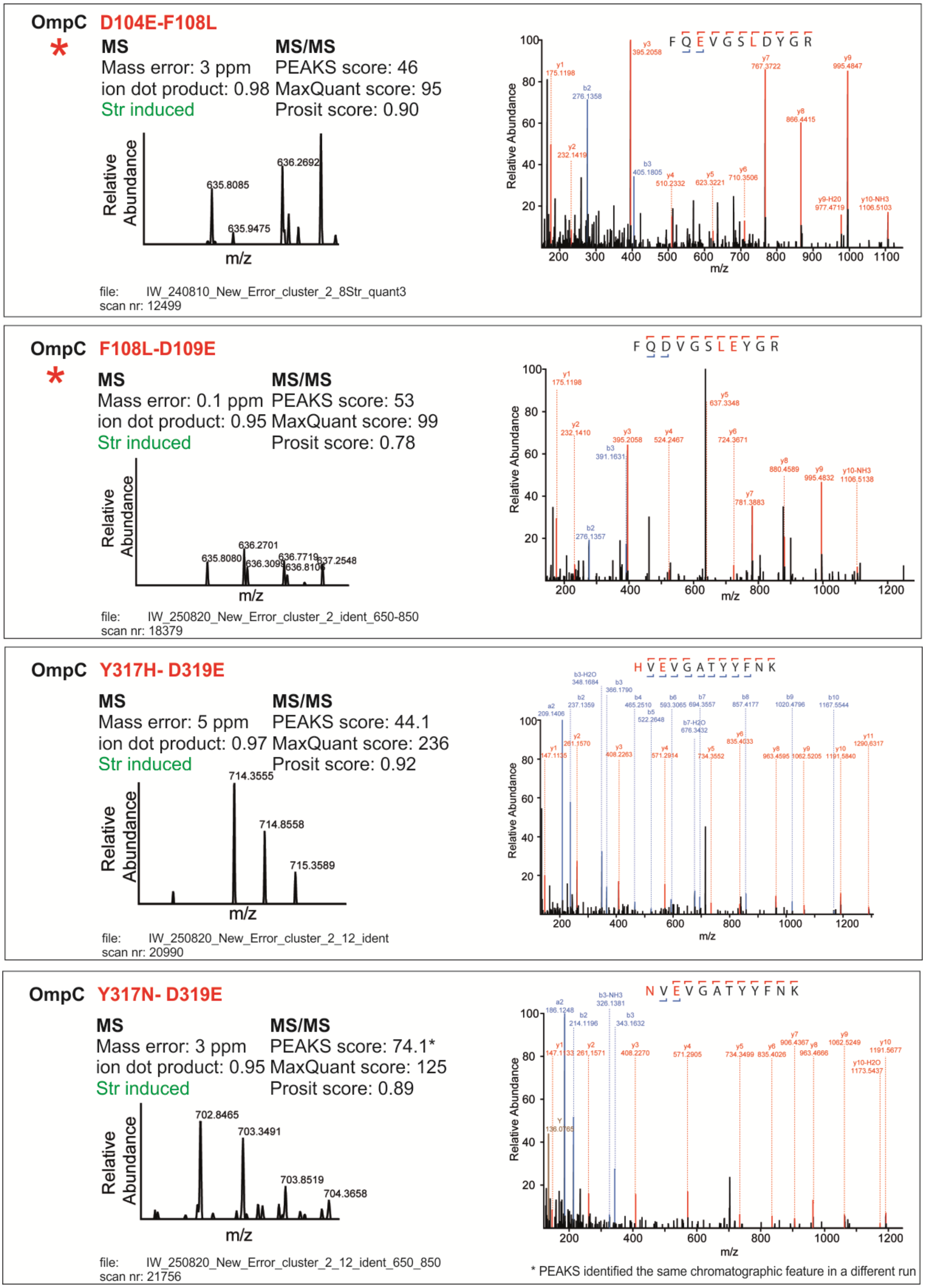
OmpC D104E-F108L
*
MS
Mass error: 3 ppm
ion dot product: 0.98
Str induced
MS/MS
PEAKS score: 46
MaxQuant score: 95
Prosit score: 0.90
Relative Abundance
m/z
635.8085
635.9475
636.2692
F Q E V G S L D Y G R
file: IW_240810_New_Error_cluster_2_8Str_quant3
scan nr: 12499
OmpC F108L-D109E
*
MS
Mass error: 0.1 ppm
ion dot product: 0.95
Str induced
MS/MS
PEAKS score: 53
MaxQuant score: 99
Prosit score: 0.78
F Q D V G S L E Y G R
file: IW_250820_New_Error_cluster_2_ident_650-850
scan nr: 18379
OmpC Y317H- D319E
MS
Mass error: 5 ppm
ion dot product: 0.97
Str induced
MS/MS
PEAKS score: 44.1
MaxQuant score: 236
Prosit score: 0.92
714.3555
714.8558
715.3589
H V E V G A T Y Y F N K
file: IW_250820_New_Error_cluster_2_12_ident
scan nr: 20990
OmpC Y317N- D319E
MS
Mass error: 3 ppm
ion dot product: 0.95
Str induced
MS/MS
PEAKS score: 74.1*
MaxQuant score: 125
Prosit score: 0.89
702.8465
703.3491
703.8519
704.3658
N V E V G A T Y Y F N K
file: IW_250820_New_Error_cluster_2_12_ident_650_850
scan nr: 21756
* PEAKS identified the same chromatographic feature in a different run

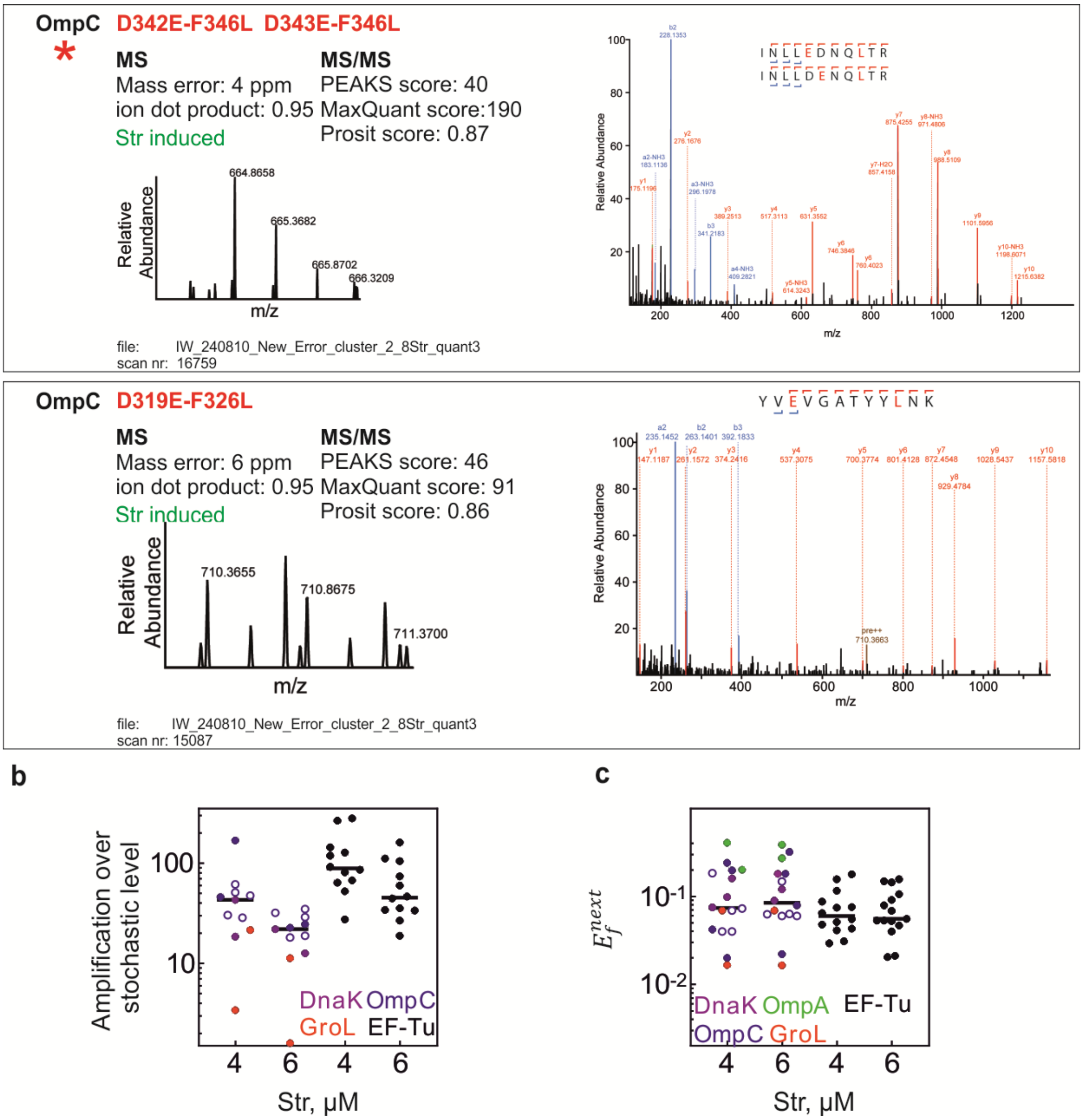

Supplementary Fig. 3: Score-based evidence and properties of error clusters detected by DDA. Related to Figs. 2 and 4

a Manually validated spectra of error clusters detected by DDA. Error clusters were identified by Spider searches in PEAKS (PEAKS scores as indicated). These candidates and those that are likely because they entail frequent single errors were further validated in MaxQuant searches (scores are indicated). Error clusters identified by MaxQuant were inspected in Skyline. Only those error clusters that were induced by Str treatment, had a mass error < 6 ppm, an idotp > 0.85, and a Prosit score > 0.75 were considered. MS/MS spectra: y-ions in red; b-ions in blue, and internal fragments in ochre. Red asterisk: Chimeric spectra of co-eluting peptides with isobaric error clusters.

b Error amplification of error clusters in different proteins. The first and the second error and the respective error cluster were quantified by LFQ. Mean values of three

technical replicates are plotted (*n*=3). The median of the amplification values is indicated as horizontal line. Different pass criteria for error clusters in the analysis of error amplification and E_f^{next} explain different numbers of clusters in b and c (see Methods). Notably, amplification is higher at lower Str concentration. For quantification of co-eluting error clusters that involve identical misreading events (open circles), equal contributions of regioisomers into the MS1 signal were assumed (see Methods, Source data file).

c E_f^{next} of error clusters in different proteins. E_f^{next} values represent the mean of three technical replicates (*n*=3) and their median is indicated as horizontal line. E_f^{next} values are independent of the Str concentration.

a

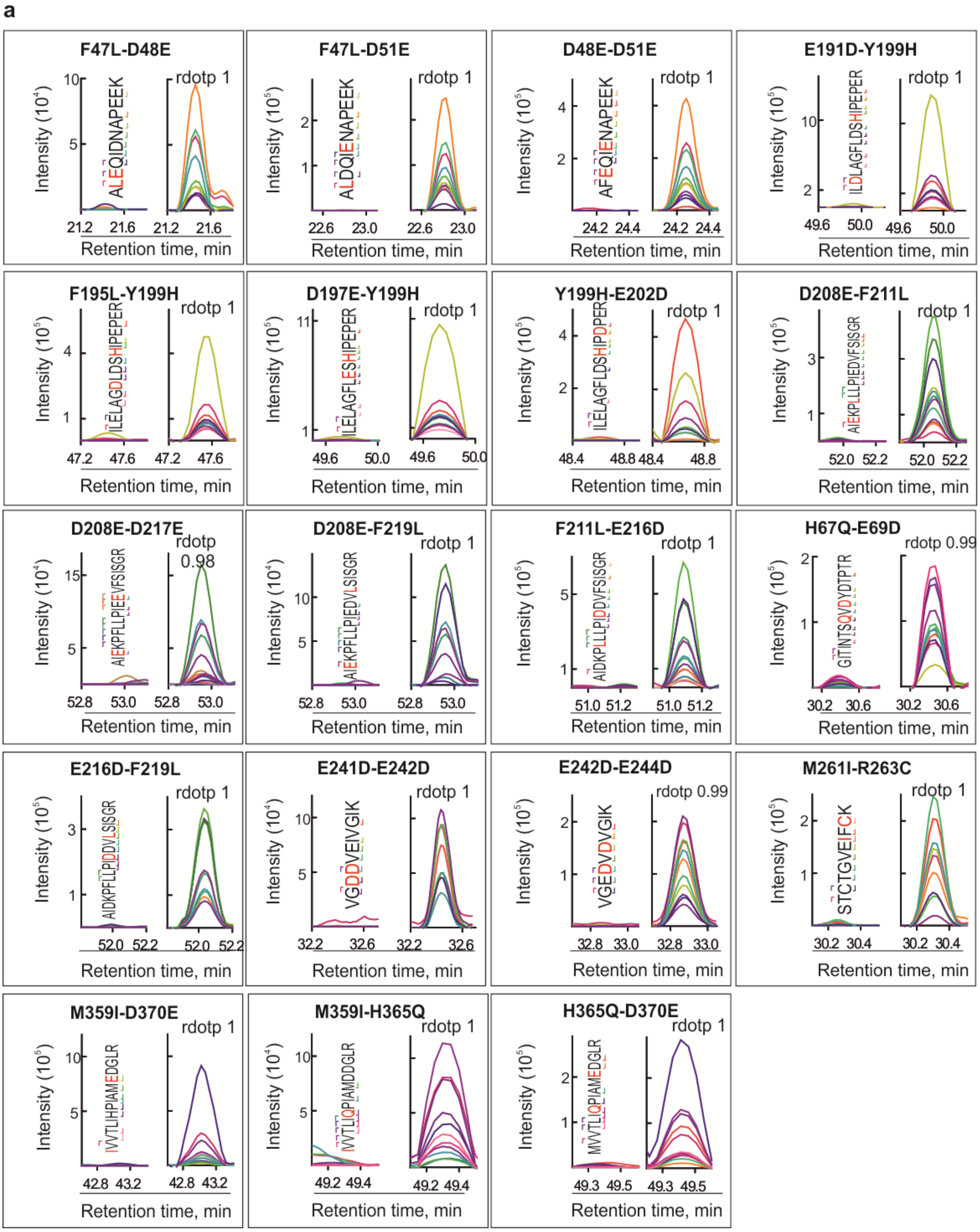

F47L-D48E
F47L-D51E
D48E-D51E
E191D-Y199H
F195L-Y199H
D197E-Y199H
Y199H-E202D
D208E-F211L
D208E-D217E
D208E-F219L
F211L-E216D
H67Q-E69D
E216D-F219L
E241D-E242D
E242D-E244D
M261I-R263C
M359I-D370E
M359I-H365Q
H365Q-D370E
rdotp 1
rdotp 0.98
rdotp 0.99
Retention time, min
ALEQIDNAPEEK
ALDQIENAPEEK
AFEQIENAPEEK
ILDLAGFLDSHIPEPER
ILELAGDLDSHIPEPER
ILELAGFLESHIPEPER
ILELAGFLDSHIPDPER
AIEKPLLLPIEDVFSISGR
AIEKPFLLPIEEVFSISGR
AIEKPFLLPIEDVLSISGR
AIDKPLLLPIDDVFSISGR
GITINTSQVDYDTPTR
AIDKPFLLPIDDVLSISGR
VGDDVEIVGIK
VGEDVDVGIK
STCTGVEIFCK
IVVTLIHPIAMEDGLR
IVVTLIQPIAMDDGLR
MVVTLIQPIAMEDGLR

b

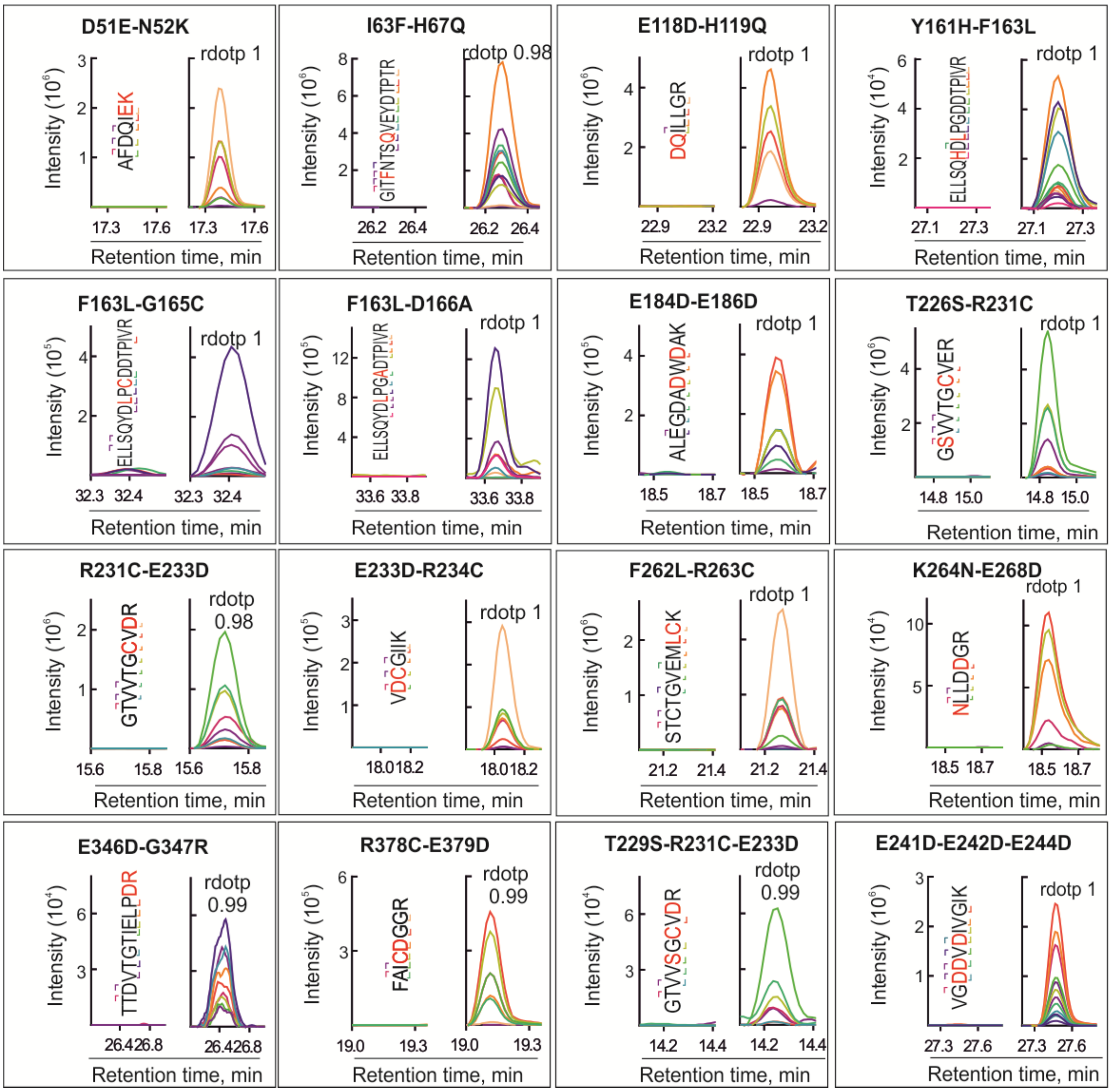
D51E-N52K
rdotp 1
AFDQIEK
Intensity (10⁶)
I63F-H67Q
rdotp 0.98
GITFNTSQVEYDTPTR
E118D-H119Q
rdotp 1
DQILLGR
Y161H-F163L
rdotp 1
ELLSQHDLPGDDTPIVR
Intensity (10⁴)
F163L-G165C
rdotp 1
ELLSQYDLPCDDTPIVR
Intensity (10⁵)
F163L-D166A
rdotp 1
ELLSQYDLPGADTPIVR
E184D-E186D
rdotp 1
ALEGDADWDAK
T226S-R231C
rdotp 1
GSWTGCVER
R231C-E233D
rdotp 0.98
GTWTGCVDR
E233D-R234C
rdotp 1
VDCGIIK
F262L-R263C
rdotp 1
STCTGVEMLCK
K264N-E268D
rdotp 1
NLLDDGR
E346D-G347R
rdotp 0.99
TTDVTGTIELPDR
R378C-E379D
rdotp 0.99
FAICDGGR
T229S-R231C-E233D
rdotp 0.99
GTWSGCVDR
E241D-E242D-E244D
rdotp 1
VGDDVDIVGIK
Retention time, min

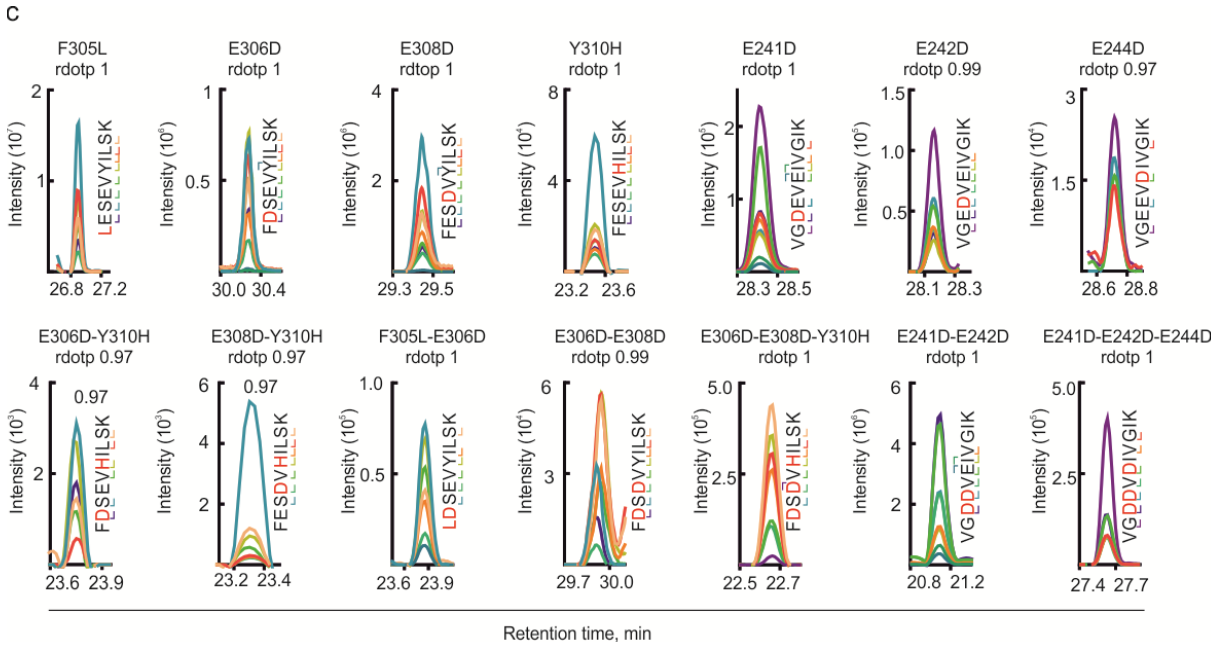

Supplementary Fig. 4: Validation of error clusters by PRM. Related to Figs. 2 and 4A

a Detected in EF-Tu peptides from the *E. coli* MG1655 lysate. Error clusters were targeted in the absence (left) and presence (right) of Str (8 µM). Error clusters were not detected in the cultures not treated with Str.

b Detected after enrichment by QRAS. EF-Tu was purified under native conditions from *E. coli* MG1655 cells grown in the absence (left) or presence (right) of Str. Target peptides were enriched from identical amounts of EF-Tu. Amounts of injected sample were equilibrated by injecting same amounts of AQUA peptide.

c Detected after enrichment and absolute quantification by QRAS (Fig. 4a).

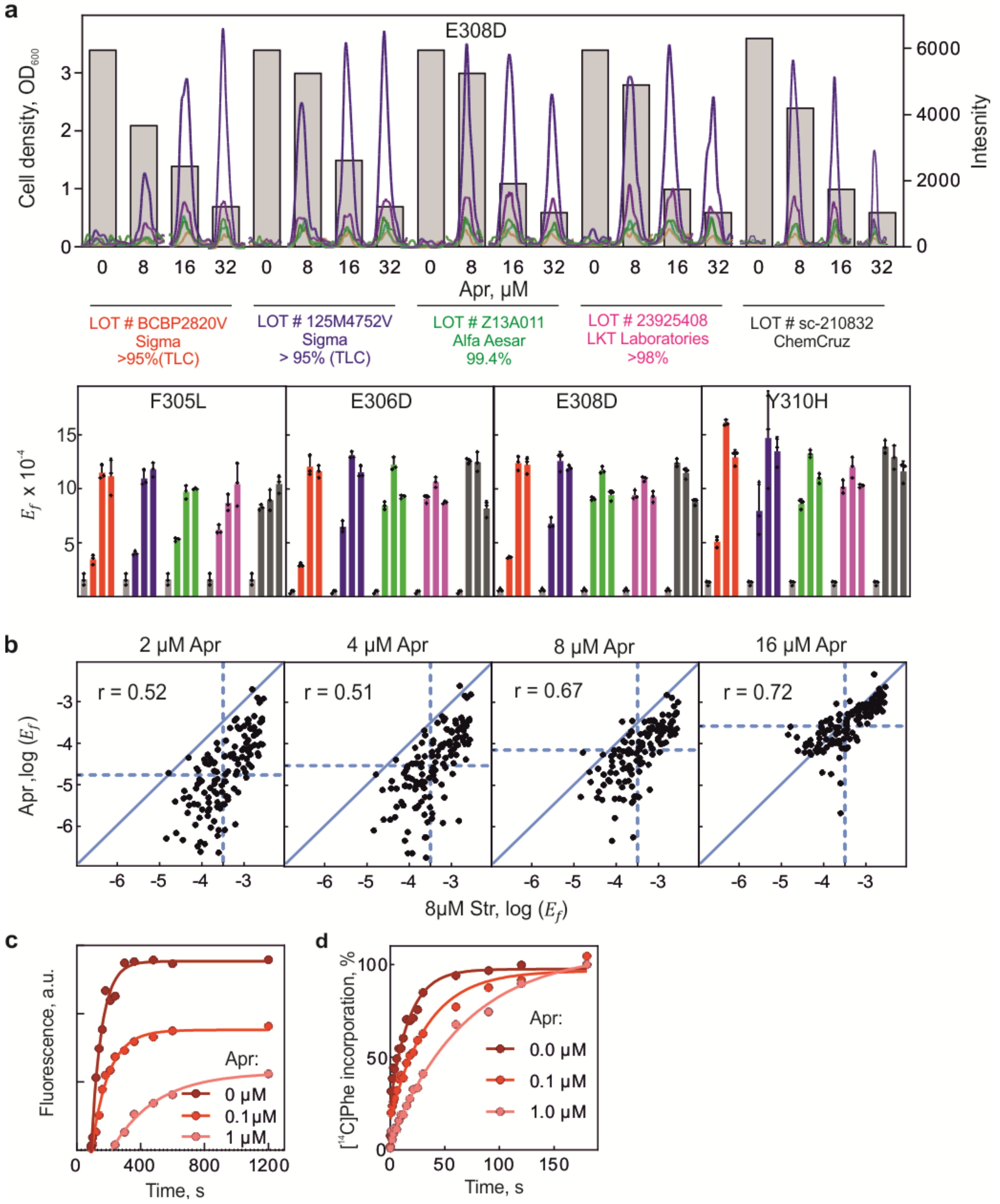

Supplementary Fig. 5: Effect of Apr on misreading and translation. Related to Fig. 3

a Comparison of Apr batches from different vendors. Upper panel: Growth inhibition (gray bars) and appearance of EF-Tu peptides with the E308D error shown as E308D SRM transitions (y5, purple; y6, green; y7, turquoise; y8, blue; y9, orange). Lower panel: Absolute quantification of selected amino acid substitutions by SRM. Shown are

means ± SD of three technical replicates (*n*=3). Color code represents vendors; Apr concentrations used for each group of bars are as in the upper panel.

b Comparative analysis of the error landscape of Str and Apr. Pearson coefficients are shown as insets.

c Time courses of the *in-vitro* synthesis of SlyD in the presence of different Apr concentrations.

d Time courses of *in-vitro* poly(Phe) synthesis in the presence of different Apr concentrations.

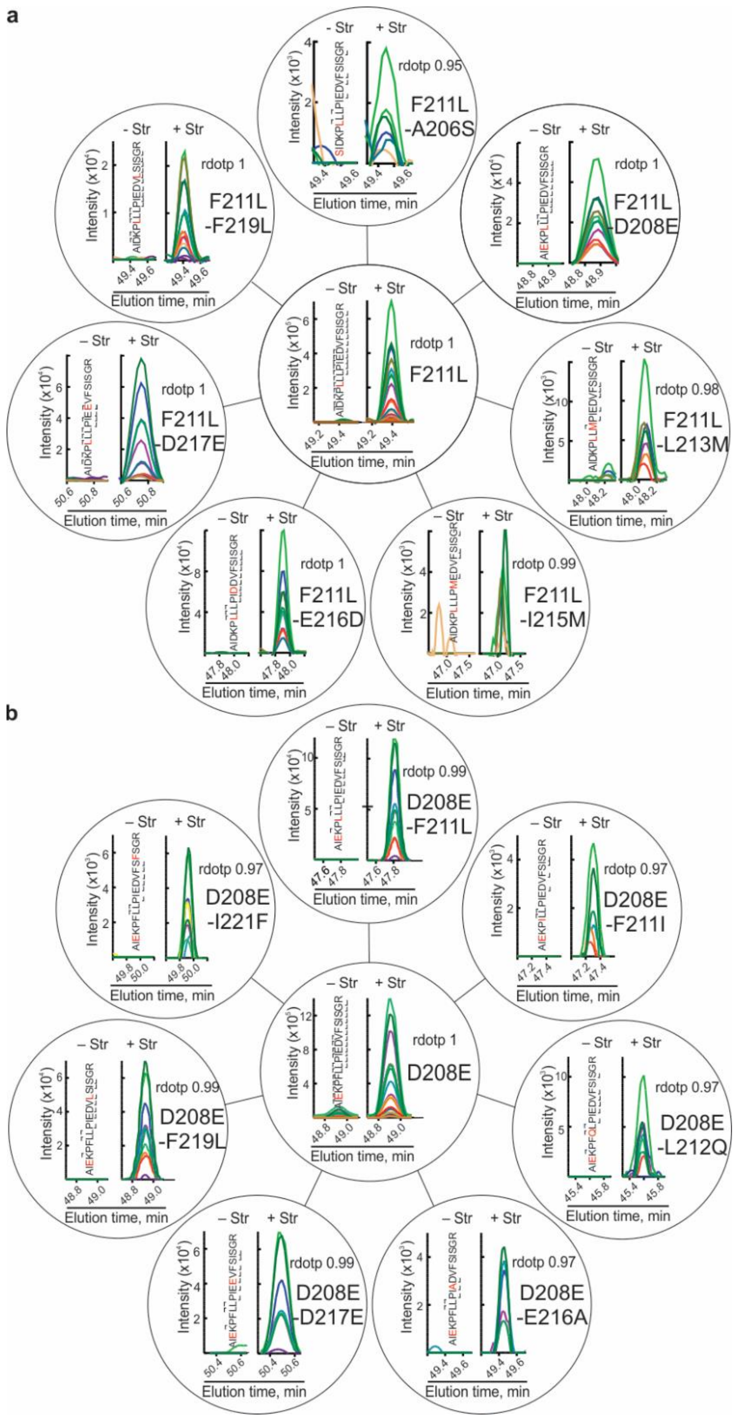

Supplementary Fig. 6: Preference for codon mismatch types in error clusters involving F211L (a) or D208E (b). Related to Fig. 4

Peptides with error clusters were targeted by PRM using the corresponding isotope-labeled reference peptides and quantified by LFQ. Identical amounts Str-treated and untreated cells were analyzed. For relative quantification shown in Fig. 4e,f, analogous sets of peptide fragment ion elution profiles of D208E/F211L and of each error cluster were integrated to determine the relative contribution of each cluster.

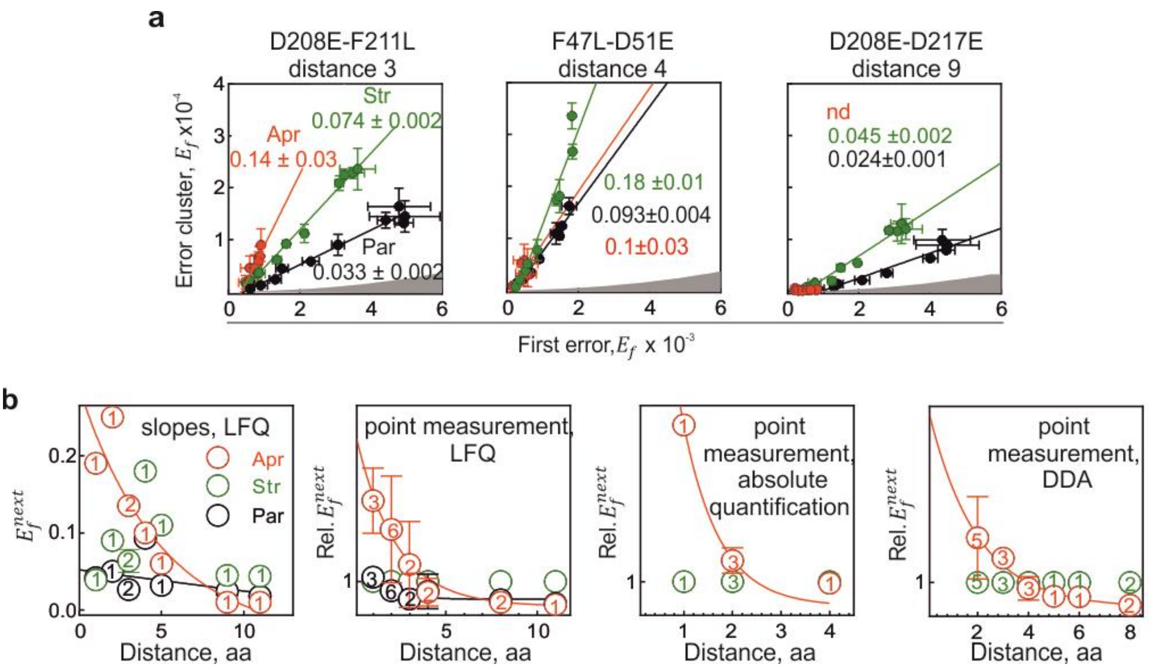

Supplementary Fig. 7: Distance dependence of error cluster formation. Related to Fig. 5

a E_f^{next} from AGA titrations. Shown are means ± SD of three technical replicates (*n*=3). D208E-F211L (Str) replotted from Fig. 4b. Expected stochastic level of error clusters are indicated as gray areas.

b Distance dependence of E_f^{next} accessed by different experimental approaches (merged in Fig. 5b). Numbers inside circles indicate the number of clusters; each value represents the mean of three technical replicates (*n*=3). When more than one cluster per distance was considered, means ± SD are shown. Panels from left to right show:

Panel 1: E_f^{next} values determined as slopes from linear concentration dependences (LFQ, data from Figs. 4d, 5a and Supplementary Fig. 7a) plotted as a function of distance between the two error positions. For long sequences, no error clusters could be observed in the presence of Apr. Thus, upper limits for the error cluster formation were imputed based on integrations over the noise of the corresponding elution time.

Panel 2: E_f^{next} values determined at individual AGA concentrations by LFQ (from Supplementary Fig. 8) plotted as a function of distance between the two error positions. Number of inspected clusters is indicated in the circles; error bars represent the standard deviation of the mean for the E_f^{next} values of different error clusters. The E_f^{next} values were normalized to that of Str, which is almost not distance-dependent and was set to 1 (see Methods).

Panel 3: E_f^{next} determined by absolute quantification after QRAS (data from Fig. 4a). The E_f^{next} were normalized to that of Str, which is essentially independent of the distance. Panel 4, E_f^{next} values derived from DDA data (from Fig. 3 a,b) plotted as a function of distance between the two error positions. The E_f^{next} values were normalized to that of Str value.

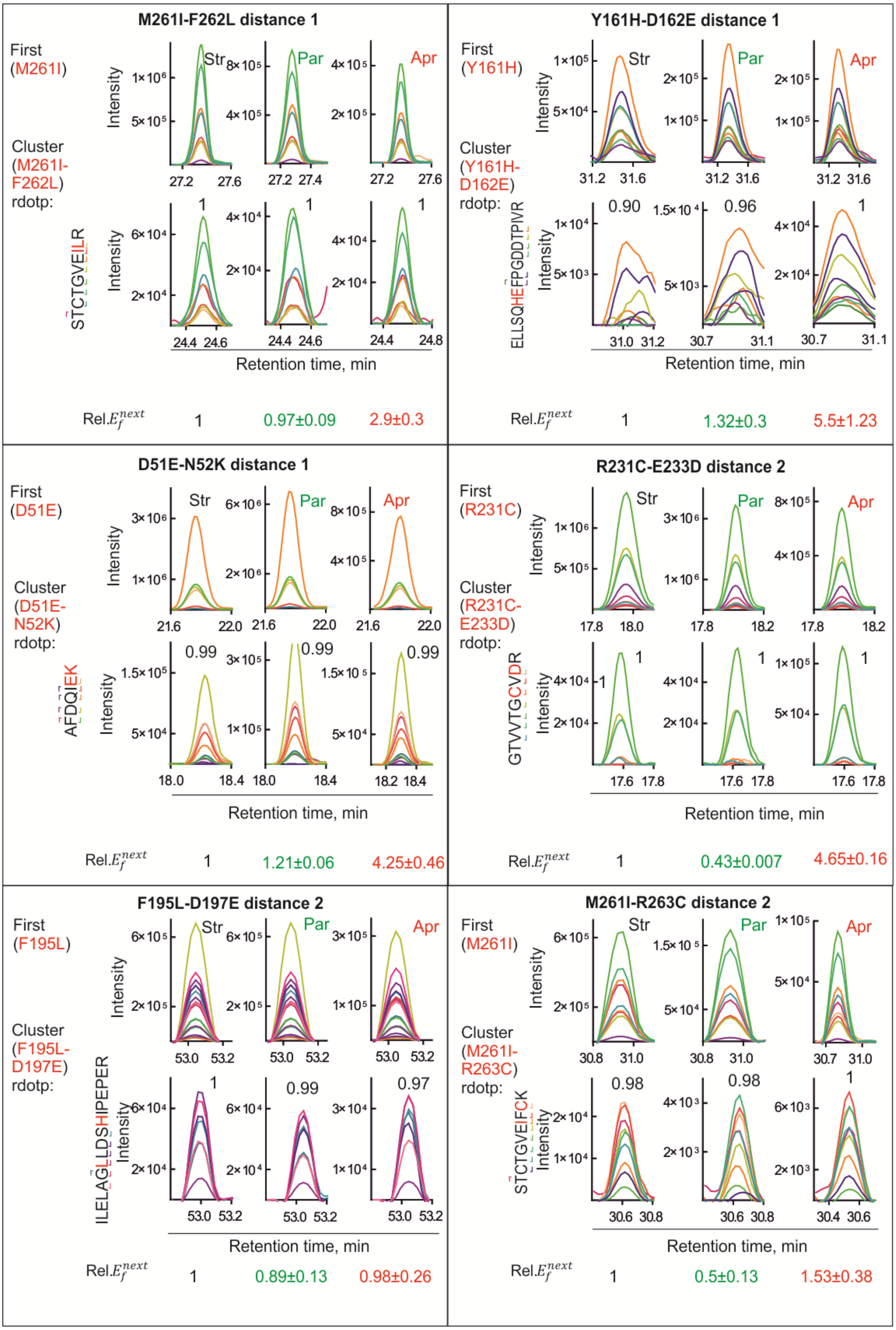
M261I-F262L distance 1
First (M261I)
Cluster (M261I-F262L) rdotp:
STCTGVEILR
Str
Par
Apr
Intensity
Retention time, min
Rel.E_f^{next} 1 0.97±0.09 2.9±0.3
Y161H-D162E distance 1
First (Y161H)
Cluster (Y161H-D162E) rdotp:
ELLSQHEFPGDDTPIVR
0.90 0.96 1
Rel.E_f^{next} 1 1.32±0.3 5.5±1.23
D51E-N52K distance 1
First (D51E)
Cluster (D51E-N52K) rdotp:
AFDQIEK
0.99 0.99 0.99
Rel.E_f^{next} 1 1.21±0.06 4.25±0.46
R231C-E233D distance 2
First (R231C)
Cluster (R231C-E233D) rdotp:
GTVVTGCVDR
1 1 1
Rel.E_f^{next} 1 0.43±0.007 4.65±0.16
F195L-D197E distance 2
First (F195L)
Cluster (F195L-D197E) rdotp:
ILELAGLLDSHIPEPER
1 0.99 0.97
Rel.E_f^{next} 1 0.89±0.13 0.98±0.26
M261I-R263C distance 2
First (M261I)
Cluster (M261I-R263C) rdotp:
STCTGVEIFCK
0.98 0.98 1
Rel.E_f^{next} 1 0.5±0.13 1.53±0.38

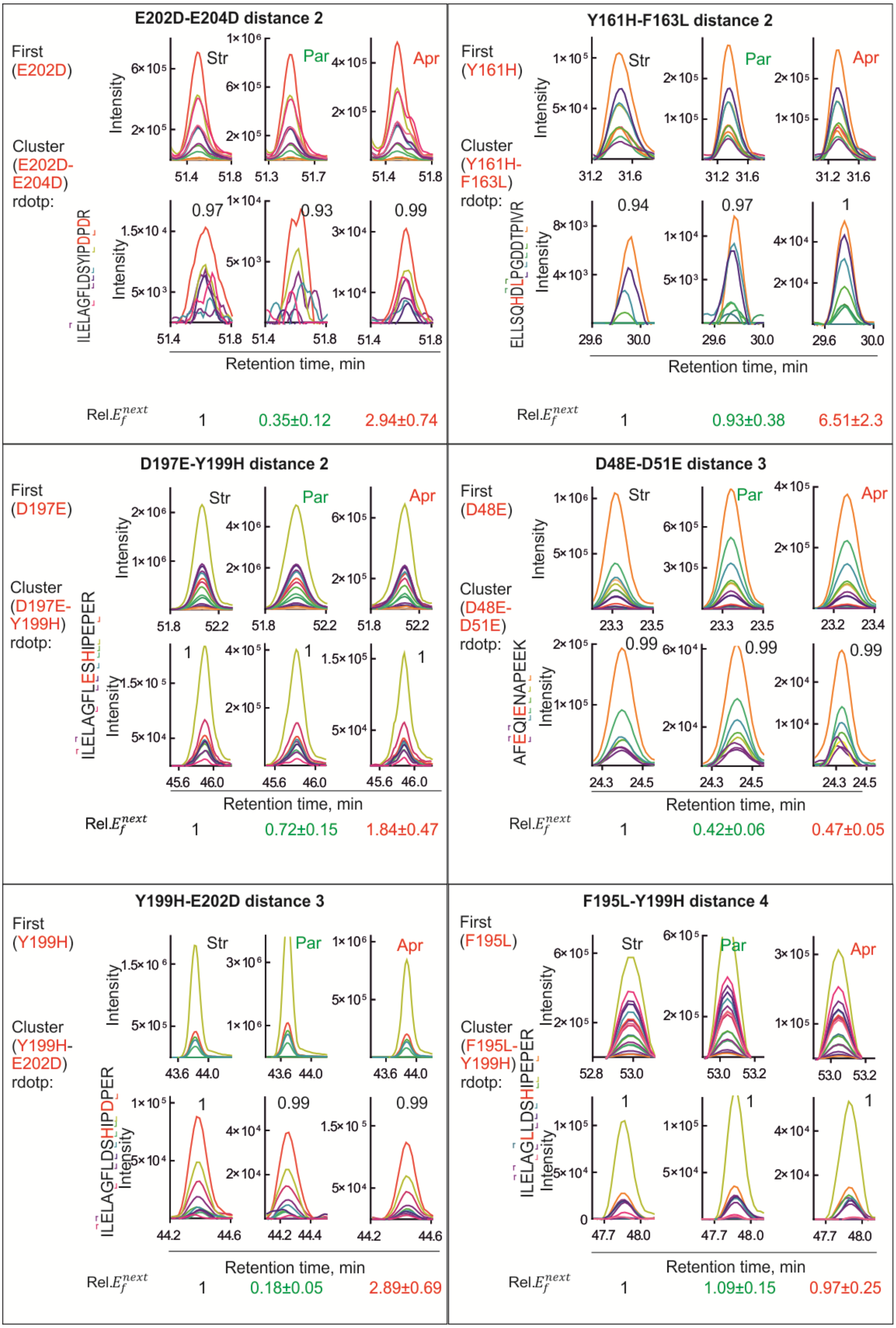
E202D-E204D distance 2
First (E202D)
Cluster (E202D-E204D)
rdotp:
ILELAGFLDSYIPDPDR
Str
Par
Apr
0.97
0.93
0.99
Retention time, min
Intensity
Rel.E_f^{next}
1
0.35±0.12
2.94±0.74
Y161H-F163L distance 2
First (Y161H)
Cluster (Y161H-F163L)
rdotp:
ELLSQHDLPGDDTPIVR
0.94
0.97
1
Rel.E_f^{next}
1
0.93±0.38
6.51±2.3
D197E-Y199H distance 2
First (D197E)
Cluster (D197E-Y199H)
rdotp:
ILELAGFLESHIPEPER
1
1
1
Rel.E_f^{next}
1
0.72±0.15
1.84±0.47
D48E-D51E distance 3
First (D48E)
Cluster (D48E-D51E)
rdotp:
AFEQIENAPEEK
0.99
0.99
0.99
Rel.E_f^{next}
1
0.42±0.06
0.47±0.05
Y199H-E202D distance 3
First (Y199H)
Cluster (Y199H-E202D)
rdotp:
ILELAGFLDSHIPDPER
1
0.99
0.99
Rel.E_f^{next}
1
0.18±0.05
2.89±0.69
F195L-Y199H distance 4
First (F195L)
Cluster (F195L-Y199H)
rdotp:
ILELAGLLDSHIPEPER
1
1
1
Rel.E_f^{next}
1
1.09±0.15
0.97±0.25

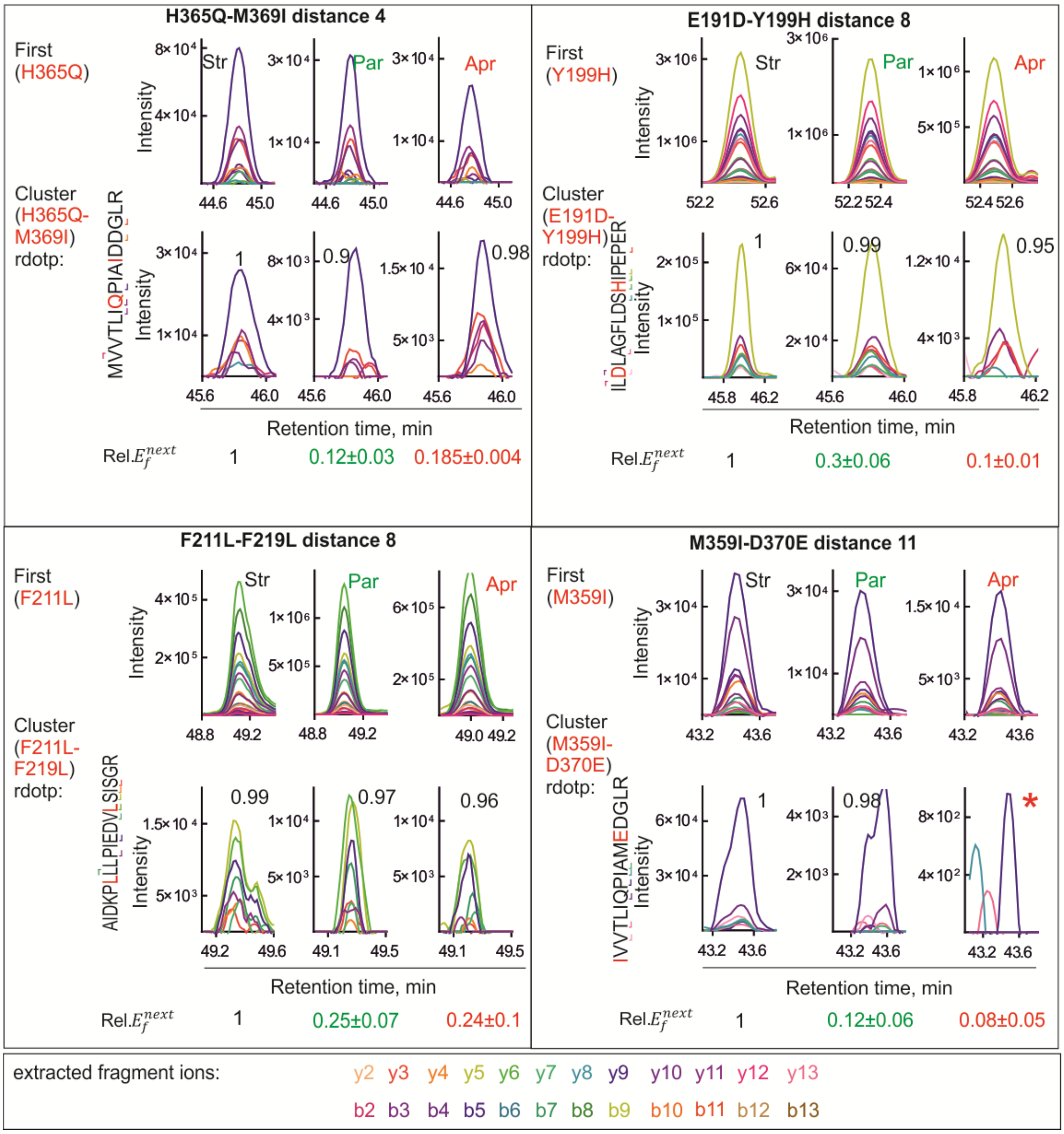

Supplementary Fig. 8: Pseudo transitions used for quantification of distance-dependence of error clusters. Related to Fig. 5

Error clusters are induced by AGA treatment (Str, 12 µM; Apr, 16 µM; Par, 8 µM). Peptides are detected by PRM and quantified by LFQ. E_f^{next} values are normalized to the Str values. E_f^{next} are means ± SD of three technical replicates. Results are replotted in Fig. 5b (left panel) and Supplementary Fig. 7 (panel 2).

Supplementary References

1. Cox J., Hein M. Y., Luber C. A., Paron I., Nagaraj N., Mann M. Accurate proteome-wide label-free quantification by delayed normalization and maximal peptide ratio extraction, termed MaxLFQ. *Mol. Cell. Proteomics* **13**, 2513-2526 (2014).

2. Tyanova S.*, et al.* The Perseus computational platform for comprehensive analysis of (prote)omics data. *Nat. Methods* **13**, 731-740 (2016).
