# Supplementary material for: Translation error clusters induced by aminoglycoside antibiotics
Source: Nat Commun. 2021 Mar 23;12:1830. doi: 10.1038/s41467-021-21942-6 (PMC7987974; doi:10.1038/s41467-021-21942-6)
Supplement: Supplementary file 2 — Reporting Summary [file 41467_2021_21942_MOESM2_ESM.pdf]

nature research

Corresponding author(s): Ingo Wohlgemuth
Marina Rodnina
Henning Urlaub

Last updated by author(s): 2021.01.20

Reporting Summary

Nature Research wishes to improve the reproducibility of the work that we publish. This form provides structure for consistency and transparency in reporting. For further information on Nature Research policies, see our Editorial Policies and the Editorial Policy Checklist.

Statistics

For all statistical analyses, confirm that the following items are present in the figure legend, table legend, main text, or Methods section.

n/a	Confirmed	
☐	☒	The exact sample size (*n*) for each experimental group/condition, given as a discrete number and unit of measurement
☐	☒	A statement on whether measurements were taken from distinct samples or whether the same sample was measured repeatedly
☐	☒	The statistical test(s) used AND whether they are one- or two-sided<br>*Only common tests should be described solely by name; describe more complex techniques in the Methods section.*
☒	☐	A description of all covariates tested
☐	☒	A description of any assumptions or corrections, such as tests of normality and adjustment for multiple comparisons
☐	☒	A full description of the statistical parameters including central tendency (e.g. means) or other basic estimates (e.g. regression coefficient) AND variation (e.g. standard deviation) or associated estimates of uncertainty (e.g. confidence intervals)
☐	☒	For null hypothesis testing, the test statistic (e.g. *F*, *t*, *r*) with confidence intervals, effect sizes, degrees of freedom and *P* value noted<br>*Give P values as exact values whenever suitable.*
☒	☐	For Bayesian analysis, information on the choice of priors and Markov chain Monte Carlo settings
☐	☒	For hierarchical and complex designs, identification of the appropriate level for tests and full reporting of outcomes
☐	☒	Estimates of effect sizes (e.g. Cohen's *d*, Pearson's *r*), indicating how they were calculated

Our web collection on statistics for biologists contains articles on many of the points above.

Software and code

Policy information about availability of computer code

Data collection	The commercial software of Thermo Fisher mass spectrometers was used for data aquisition (see Source data file). Fluorescent peptides were detected using Starion IR/FLA-9000 scanner (FujiFilm).
Data analysis	DDA data was analyzed in MaxQuant (version 1.5.5.1, 1.6.0.1, 1.6.5.0) or PEAKS (version 10.5). Proteomics results were analyzed in Perseus (version 1.6.5.0) or Skyline (4.2.0.19009, 20.1.1.158 and 3.5). Targeted data was analyzed in Skyline. Multigauge software of the Fujifilm FLA-9000 fluorescence scanner was used to quantify the translation time courses. Data were visualized in GraphPadPrism (version 8.3) and CorelDRAW (version 18.1.0.661).

For manuscripts utilizing custom algorithms or software that are central to the research but not yet described in published literature, software must be made available to editors and reviewers. We strongly encourage code deposition in a community repository (e.g. GitHub). See the Nature Research guidelines for submitting code & software for further information.

Data

Policy information about availability of data

All manuscripts must include a data availability statement. This statement should provide the following information, where applicable:
- Accession codes, unique identifiers, or web links for publicly available datasets
- A list of figures that have associated raw data
- A description of any restrictions on data availability

A detailed report on the data structure and availability is provided in the Source data file.
MS data are deposited to the ProteomeXchange Consortium. All DDA data were deposited via PRIDE partner repository:
1. All DDA data for EF-Tu:
Project Name: Translation Error Clusters Induced by Aminoglycoside Antibiotics

Website: https://www.ebi.ac.uk/pride/
ProteomeXchange ID: PXD019188

2. Additional data on the identification of error clusters in other proteins:
Project Name: Translation Error Clusters Induced by Aminoglycoside Antibiotics
ProteomeXchange ID: PXD022098

We deposited analysis steps of the DDA data and targeted MS data via the PanoramaPublic partner repository.
Access URL (https://panoramaweb.org/Error-cluster.url)
Reserved ProteomeXchange ID: PXD019328

These data provide evidence to substantiate all conclusions. The residual raw data can be provided upon reasonable request.

Field-specific reporting

Please select the one below that is the best fit for your research. If you are not sure, read the appropriate sections before making your selection.

[x] Life sciences [ ] Behavioural & social sciences [ ] Ecological, evolutionary & environmental sciences

For a reference copy of the document with all sections, see nature.com/documents/nr-reporting-summary-flat.pdf

Life sciences study design

All studies must disclose on these points even when the disclosure is negative.

Sample size	We report the existence of AGA-induced error clusters and validated 96 clusters in 9 proteins of E. coli. As we report a qualitatively new phenomenon, a sample size calculation was not necessary.
Data exclusions	In general, no data was excluded. Especially for the characterization of error clusters all analyzed examples are reported. In Figures 4b,d,5a,7a samples from low AGA concentrations for which no error clusters were detectable by PRM were excluded from linear fitting.
Replication	Experiments that led to quantitative conclusions were repeated (as indicated in figure legends). Biological replicates include individual cell growth, drug treatment, sample processing, data acquisition and analysis. Technical replicates are based on repeatedly analyzed samples. All findings could be reproduced and no replicate led to deviating conclusions.
Randomization	Sample randomerization is not necessary (see also Blinding). Because aminoglycosides increase the frequency of errors over orders of magnitude, blinding and randomerization would hinder an efficient control of chromatographic carry over.
Blinding	Samples were not blinded. As we use the misreading inducing property of aminoglycosides as intensity-based filter in our analysis, blinding is not compatible with our experimental setup. Our exploratory study reveals qualitatively new phenomena, thus an expectancy or confirmation bias is excluded. All conclusions are validated by different experimental approaches.

Reporting for specific materials, systems and methods

We require information from authors about some types of materials, experimental systems and methods used in many studies. Here, indicate whether each material, system or method listed is relevant to your study. If you are not sure if a list item applies to your research, read the appropriate section before selecting a response.

Materials & experimental systems

n/a	Involved in the study
[x]	[ ] Antibodies
[x]	[ ] Eukaryotic cell lines
[x]	[ ] Palaeontology and archaeology
[x]	[ ] Animals and other organisms
[x]	[ ] Human research participants
[x]	[ ] Clinical data
[x]	[ ] Dual use research of concern

Methods

n/a	Involved in the study
[x]	[ ] ChIP-seq
[x]	[ ] Flow cytometry
[x]	[ ] MRI-based neuroimaging
